# Therapeutic Significance of NLRP3 Inflammasome in Cancer: Friend or Foe?

**DOI:** 10.3390/ijms252413689

**Published:** 2024-12-21

**Authors:** Aliea M. Jalali, Kenyon J. Mitchell, Christian Pompoco, Sudeep Poludasu, Sabrina Tran, Kota V. Ramana

**Affiliations:** Department of Biomedical Sciences, Noorda College of Osteopathic Medicine, Provo, UT 84606, USA

**Keywords:** cancer, NLRP3, inflammasome, innate immunity, IL-1b, caspase-1

## Abstract

Besides various infectious and inflammatory complications, recent studies also indicated the significance of NLRP3 inflammasome in cancer progression and therapy. NLRP3-mediated immune response and pyroptosis could be helpful or harmful in the progression of cancer, and also depend on the nature of the tumor microenvironment. The activation of NLRP3 inflammasome could increase immune surveillance and the efficacy of immunotherapy. It can also lead to the removal of tumor cells by the recruitment of phagocytic macrophages, T-lymphocytes, and other immune cells to the tumor site. On the other hand, NLRP3 activation can also be harmful, as chronic inflammation driven by NLRP3 supports tumor progression by creating an environment that facilitates cancer cell proliferation, migration, invasion, and metastasis. The release of pro-inflammatory cytokines such as IL-1β and IL-18 can promote tumor growth and angiogenesis, while sustained inflammation may lead to immune suppression, hindering effective anti-tumor responses. In this review article, we discuss the role of NLRP3 inflammasome-mediated inflammatory response in the pathophysiology of various cancer types; understanding this role is essential for the development of innovative therapeutic strategies for cancer growth and spread.

## 1. Introduction

The NOD-like receptor (NLR) protein family, a type of pattern recognition receptor (PRR), plays a vital role in the cellular innate immune response by identifying foreign substances, triggering an immune response, and assembling inflammasomes [1]. Within humans, there are currently 14 known NLR protein members, named NLPR1 through NLPR14, involved in various aspects of immune cell regulation [1]. Activation of NLR proteins leads to inflammatory responses mediated through nuclear factor kappa binding protein (NF-κB), mitogen activated protein kinase (MAPK), or caspase-1 activation and the release of proinflammatory cytokines [2]. Dysregulation and malfunction of NLR variants have been linked with the pathophysiology of inflammatory diseases, and there is also evidence that they may contribute to cancer progression by controlling inflammatory signaling pathways to promote cell proliferation, migration, and invasion [3,4].

**Figure 1:**
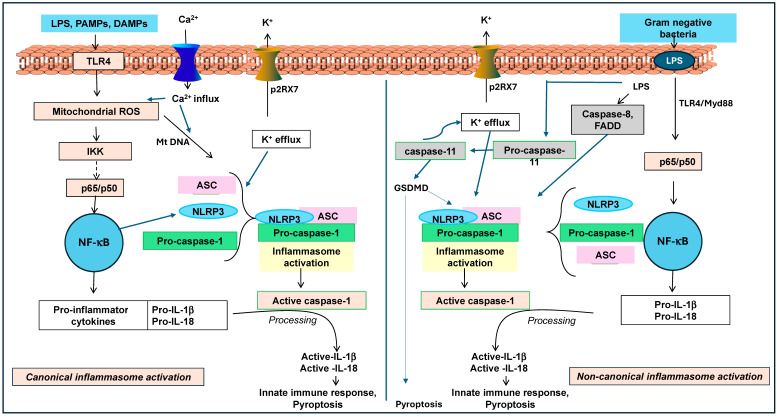
Canonical and non-canonical activation of NLRP3 inflammasome. The canonical pathway involves the activation of NLRP3 inflammasomes through signals such as mitochondrial ROS, calcium influx, and potassium efflux, leading to NF-κB activation and the production of pro-inflammatory cytokines (IL-1β and IL-18). This pathway ultimately activates caspase-1, resulting in cytokine release and pyroptosis. The non-canonical pathway involving LPS from Gram-negative bacteria triggers caspase-11, which indirectly activates NLRP3, leading to similar inflammasome responses, cytokine production, and pyroptosis. Both pathways generally play critical roles in innate immunity and inflammation.

The Nod-like receptor protein 3 (NLRP3) inflammasome is a multi-protein assembly that triggers initial innate immune responses by activating caspase-1 and releasing proinflammatory cytokines such as interleukin (IL)-1β and IL-18 in response to pathogen-associated molecular patterns (PAMPs) and damage-associated molecular patterns (DAMPs) (Figure 1) [5]. The inflammasome activation could cause a specific programmed cell death called pyroptosis. Generally, pyroptosis helps to eliminate infected or damaged cells. Thus, inflammasomes are essential for defending the body against pathogen attacks. However, their overactivation or dysfunction is linked to various inflammatory diseases, including gout, atherosclerosis, and cancer. The NLRP3 inflammasome is of particular interest, due to its ability to have both protective and damaging effects on carcinogenesis, depending on the type of cancer [6]. Further, several studies have indicated that overactivation of the NLRP3 inflammasome has been linked to cancer progression [1,4,7]. Conversely, deficiencies in the NLRP3 inflammasome have shown a potential to increase tumor burden in certain cancers, such as colitis-associated cancer, due to a lack of tumor-suppressing cytokines [6,7].

Abderrazak et al. and Leemans et al. [8,9] have demonstrated that NLPR3 inflammasome-generated IL-1β and IL-18 promote immune and inflammatory responses and accelerate tumor growth and spread. IL-1β and IL-18, generated by the NLRP3-inflammasome, could cause persistent immune and inflammatory responses by activating the generation of various other immune cell-mediated cytokines and chemokines such as tumor necrosis factor-alpha (TNF-α), IL-6, IL-4, interferon-gamma (IFN-γ), monocyte chemoattractant protein-1 (MCP-1) and melanocyte-inhibiting factor (MIF-1) leading to tumor growth and metastasis. Further, this inflammatory state can create a tumor-promoting environment by stimulating cancer cell growth and survival. In cancers such as colon and breast, the inflammatory mediators released by the NLRP3 inflammasome have been associated with increased cancerous growth and metastasis [10,11]. In some instances, IL-1β and IL-18 also contribute to the survival of the cancer cells, and that helps them spread to other organs by modulating the epithelial-to-mesenchymal transition (EMT) [12,13,14]. Therefore, inflammation is a significant risk factor for cancer development in several ways. It can cause DNA damage leading to mutations, disturb the immune balance and promote cell growth, increase the expression of growth factors that initiate angiogenesis required for tumor growth, and impair immune surveillance. Thus, chronic and uncontrolled inflammation caused by NLRP3-dependent and independent mechanisms could lead to many cancer types.

However, the NLRP3 inflammasome can also play a role in suppressing cancer through mechanisms related to immune surveillance. Further, activation of inflammasome could recruit immune cells to the tumor site, leading to the destruction of cancer cells. For instance, Wu et al. [15] have shown that the NLRP3 inflammasome can induce pyroptosis, a programmed cell death that could halt tumor growth. In addition, the inflammatory response triggered by the NLRP3 inflammasome can also stimulate anti-tumor immunity, which enhances the T-cell-mediated elimination of cancer cells [16].

The dual action of the NLRP3 inflammasome in cancer underscores its complex and context-dependent role. On one hand, NLRP3 inflammasome could act as a driver of tumor progression through chronic inflammation. On the other hand, it could support the tumor-associated immune system to promote tumor growth [7,17]. Therefore, further investigations are required to understand the mechanisms through which the NLRP3 inflammasome regulates cancer growth and progression. In this review, we discuss the possible role of NLRP3 inflammasome in the mediation of tumor growth and spread and its potential therapeutic use. We searched PubMed and Google Scholar to find articles published in the last 10 years or so, using keywords including NLRP3, inflammasome, immune response, IL-1β, IL-18, pyroptosis, melanoma, leukemia, breast cancer, colon cancer, lung cancer, and other cancer types. We included various research articles, comprehensive narrative reviews, meta-analytical studies, systematic reviews, clinical studies, and pre-clinical studies, to discuss their findings. We did not include studies on other types of inflammasomes. This narrative review article discusses only the role and therapeutic significance of the NLRP3 inflammasome in various cancers.

## 2. NLRP3 in Melanoma

Melanoma is a melanocyte tumor, and is the fifth leading cancer in the United States. Its incidence rate is rising globally, and 80% of skin cancer deaths are associated with melanoma [18]. Although melanoma incidence has been shown to be dependent on the skin type of melanin-producing cells, recent evidence suggests that mutations due to ultraviolet (UV) radiation (UVA (315–400 nm) and UVB (280–315 nm))/sun exposure are the major cause of neoplasm development [19]. Further, UV beds (indoor tanning stations) could also cause skin cells’ DNA damage and risk of developing melanoma. Specifically, mutations associated with genes such as BRAF, NRAS, KIT, and MAPK have been associated with melanoma [20,21]. Further, the melanoma subtypes and prognosis are dependent on the location of these mutations [21]. Besides genetic mutations, recent studies also indicated the significance of macrophages and other immune cell-mediated inflammatory responses as a major cause of melanoma [22,23]. In some cases, the immune cells could act as biomarkers of melanoma [24]. For example, Reschke et al. [25] have shown that tissue-resident memory T-cells could be potential biomarkers of melanoma.

Macrophage polarization into M1 and M2 phenotypes also plays a significant role in the progression of melanoma [26,27]. The M1 phenotype is pro-inflammatory, while the M2 phenotype is anti-inflammatory and pro-angiogenic, and both cell types can influence tumor progression and immune response. However, in melanoma, increased CD163+ tumor-infiltrating macrophages (M2 phenotype) lead to the loss of Melan-A expression, causing pronounced invasive tumor phenotype [28]. Further, it has been shown that complement component C3a has been identified as playing a role in melanoma progression by affecting immune cell activation and tumor promotion [29]. Similarly, a few studies have also shown that the presence of mature dendritic cells (DCs) and Langerhans cells could act as prognostic indicators [30,31]. A high number of mature DCs correlates with better patient outcomes. Further, it has been shown that pro-inflammatory cytokines and chemokines like IL-1β, IL-6, MCP-1, and TNF-α also play a major role in the inflammation-mediated pathogenesis of melanoma [32]. In addition, NLRP3-mediated immune and inflammatory responses also play an important role in the growth and progression of melanoma, as well as its resistance to immunotherapy. For example, Chow et al. [33] have demonstrated that NLRP3 deficiency prevents methylcholanthrene-induced sarcoma and metastasis in a mouse model by natural killer cell-mediated regulation of immune responses. Similarly, Drexler et al. [34]. have shown that IL-1r and caspase-1 deficient mice are partially protected from epithelial skin cancer. Further, inhibition of NLRP3 inflammasome by thymoquinone prevents mouse and human melanoma cell migration. The NLRP3 inflammasome has been associated with both positive and negative predictive outcomes in melanoma patients. For example, elevated NLRP3 gene-expression scores have been linked to a favorable prognosis [35]. However, other studies have reported that NLRP3 inflammasome activation and the production of IL-1β and IL-18 can promote melanoma growth [36,37]. Further, NLRP3 and IL-1β have been shown to be overexpressed in melanoma samples, and targeting tumor-associated NLRP3 prevents melanoma growth by reducing the expansion of myeloid-derived suppressor cells (MDSCs) [38].

The NLRP3 inflammasome has also been shown to establish a pulmonary metastatic niche and drive disease hyper-progression in response to immunotherapy [39]. Okamoto et al. [40] have also been implicated in promoting late-stage melanoma progression through the cleavage of caspase-1 and active IL-1β. On the other hand, Manica et al. [41] have shown that curcumin prevents cutaneous melanoma by downregulation of NLRP3 activation and decreased IL-1β and IL-18 secretion. Similarly, rosmarinic acid has been shown to prevent the growth and migration of human metastatic melanoma cells by preventing the NLRP3 expression [42]. Moreover, combining anti-programmed cell death-(PD)-1 therapy with NLRP3 inhibition suppressed primary melanoma progression and distant melanoma metastases more effectively than anti-PD-1 monotherapy [38].

**Figure 2:**
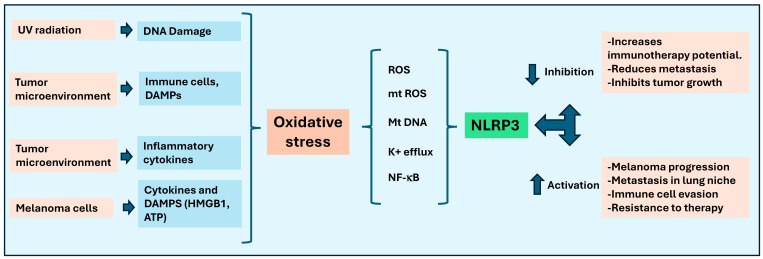
Significance of NLRP3 inflammasome in the melanoma progression. Various factors such as UV radiation, tumor microenvironment, and melanoma cells contribute to oxidative stress and cause DNA damage, immune cell activation, and cytokine release. Oxidative stress, in turn, triggers reactive oxygen species (ROS), mitochondrial DNA damage, potassium efflux, and NF-κB activation, which influence the activation of the NLRP3 inflammasome. NLRP3 activation promotes melanoma progression, metastasis, immune evasion, and therapy resistance. In contrast, inhibition of NLRP3 could enhance immunotherapy, inhibit tumor growth, and reduce metastasis.

Thus, current findings suggest a dual role for NLRP3 in melanoma, where its activation can promote or inhibit tumor growth and progression, depending on the context (Figure 2). In the context of immunotherapy, the tumor-intrinsic NLRP3-heatshock protein 70 (HSP70) toll-like receptor 4 (TLR4) axis has been associated with disease hyper-progression and survival in patients undergoing anti-PD-1 immunotherapy [39]. For example, increased NLRP3 activity in melanoma due to immunotherapy has been shown to regulate the expressions of PD-L1 and Cd155 in the tumor microenvironment [43,44]. Thus, these studies indicate that NLRP3 is involved in the immunotherapy resistance to melanoma. However, additional pre-clinical and clinical studies are required to clearly understand the significant role of NLRP3 in melanoma progression, as well as providing resistance to immunotherapy.

## 3. NLRP3 in Leukemia

Leukemia is a blood-related cancer characterized by the neoplastic transformation of hematopoietic progenitor cells and their infiltration into the bone marrow [45]. According to the National Cancer Institute, as of 2024, leukemia continues to be a significant health concern, with varying incidence and mortality rates for different types of disease. In the United States, it is estimated that there will be 62,770 new cases of leukemia and approximately 23,670 deaths from the disease (https://seer.cancer.gov/statfacts/html/leuks.html (accessed on 22 August 2024)). The most common subtypes of leukemia are acute myeloid leukemia (AML) and chronic lymphocytic leukemia (CLL), with CLL accounting for more mortality. Leukemia can affect people of all ages but is most common in children and older adults. When compared to females, males generally have higher incidence rates. Acute lymphoblastic leukemia (ALL) is more prevalent in children, while AML is more common in adults. CLL and chronic myeloid leukemia (CML) could primarily affect older individuals [45]. Moreover, inherited mutations, environmental factors, age, and unhealthy lifestyle choices are the major risk factors for the development of leukemia.

Further, the immune and inflammatory responses could also play a significant role in leukemia progression, by providing immune surveillance [46,47]. However, leukemia cells can also evade immune detection and chronic inflammation and create a supportive environment for leukemia growth [47]. Recent studies have shown that advanced treatment strategies such as targeted therapies and immunotherapies have improved survival rates, particularly for childhood leukemia [48,49,50]. Nevertheless, significant geographical disparities in incidence and outcomes highlight the necessity of early diagnosis and treatment.

Further, recent studies also indicate that NLRP3 inflammasome activation has been implicated in various types of leukemia, including myelodysplastic syndrome, myeloproliferative neoplasms, AML, CML, and ALL [51,52,53]. Activation of the NLRP3 inflammasome has been associated with promoting proliferation, inhibiting apoptosis, and increasing drug resistance in primary leukemia cells [54]. NLRP3 plays a crucial role in AML by promoting a pro-inflammatory environment through inflammasome activation, regulating cell death via pyroptosis, and aiding immune evasion by leukemic cells. Aberrant NLRP3 activity supports AML progression and survival. Wang et al. [55] have shown that genetic polymorphisms of cytokine genes IL-18 rs1946518 and IL-1β rs16944 are linked with the prognosis and survival of AML patients. Further, Zhong et al. [54] have demonstrated the significance of NLRP3 inflammasome-activated IL-1β in the AML progression. They have shown that the knockdown of NLRP3 prevents leukemia burden in an AML mouse model. This study suggested that targeting NLRP3 is beneficial in controlling AML. Liu et al. [56] have shown that NLRP3-activated bone marrow-derived cells promote Th1 cell differentiation via IL-1β secretion. This, in turn, increases IFN-γ production, induces apoptosis, and inhibits the proliferation of leukemia cells, suggesting a potential approach for AML immunotherapy. Similarly, Liu et al. [57] have demonstrated that stress worsens AML by increasing leukemic cell infiltration and reducing cell survival in a mouse model of chronic restraint stress. This is accompanied by increased NLRP3 inflammasome activation and IL-1β levels. Further, they have shown that knockdown of high-mobility group box 1 (HMGB1) protein, which is highly expressed in newly diagnosed AML patients, suppressed NLRP3 inflammasome and IL-1β expressions, which mitigated the adverse effects of stress on AML progression.

Additionally, Hamarsheh et al. [58] have demonstrated that the NLRP3 inflammasome is implicated in other hematological diseases, such as chronic myelomonocytic leukemia and juvenile myelomonocytic leukemia, particularly through the Kirsten rat sarcoma viral oncogene homologue (KRAS)/RAC1/reactive oxygen species (ROS)/NLRP3/IL-1β axis. This pathway is involved in myeloproliferation and cytopenia, and its effects are reversible in models with NLRP3 deficiency. This study also indicates that the KRAS/ROS/NLRP3/IL-1β axis is also active in AML cells, and suggests that KRAS activation leads to RCA1 activation, which, via NADPH oxidase activation and ROS generation, promote NLRP3-mediated release of innate immune cytokines. In addition, Jia et al. [59] have shown that newly diagnosed AML patients exhibited higher expression of NLRP3 inflammasome and aryl hydrocarbon receptor (AHR) in the bone marrow and peripheral blood, along with elevated IL-18 levels and an increased Th22/Th1 ratio compared to patients in complete remission and normal controls. These studies suggest that NLRP3, via AHR, could play an important role in T-helper cell differentiation and AML progression. Additionally, the NLRP3 inflammasome has been linked to the development of myeloid leukemias, such as chronic myelomonocytic leukemia (CMML), juvenile myelomonocytic leukemia (JMML), and AML, particularly in the context of KRAS mutations [60,61,62].

Glucocorticoids are one of the options for treating patients with ALL, and the treatment is based on the sensitivity and resistance of ALL cells to glucocorticoids [63]. Furthermore, NLRP3 inflammasome and caspase-1 activation have been associated with glucocorticoid resistance in leukemia cells, which has implications for treatment response and disease recurrence [64]. Interestingly, caspase-1 activation could cause glucocorticoid receptor cleavage, which leads to reduced glucocorticoid-induced transcriptional activation and enhanced glucocorticoid resistance. On the other hand, caspase-1 inhibition has been shown to restore glucocorticoid sensitivity by increasing the expression of receptors [65]. Current findings on ALL indicate that glucocorticoid resistance, linked to poorer prognosis, correlates with elevated caspsase-1 and NLRP3 expression, due to reduced methylation of their promoters in leukemia cells [64,65]. In the context of chemotherapy, the NLRP3 inflammasome has been implicated in contributing to therapy resistance in ALL. For example, Hu et al. [66] have suggested that in patients with ALL, post-chemotherapy IL-18 levels in plasma were significantly increased when compared to pre-chemotherapy levels. Chemotherapy induces upregulation of NLRP3, and interestingly, the triterpenoid analog CDDO-2P-Im (1-(2-cyano-3,12-dioxooleana-1,9(11)-dien-28-oyl)-4(-pyridin-2-yl)-1H-imidazole) inhibits NLRP3 inflammasome activation, reduces ALL cell growth in vitro, and promotes apoptosis in steroid-sensitive and steroid-resistant leukemia cells [67]. Thus, this study indicates that inhibition of NLRP3 inflammasome could reverse glucocorticoid resistance and, therefore, could improve the treatment options for ALL. In addition, genetic polymorphisms of NLRP3 inflammasome genes have been investigated for their association with pediatric ALL [68]. These studies provided new insights into the genetic basis of leukemia susceptibility and outcomes. Similarly, genetic polymorphisms associated with NLRP3 have been linked to CML [69].

**Figure 3:**
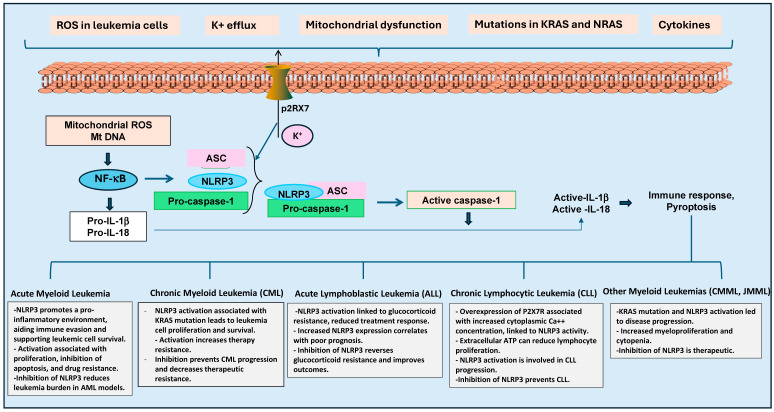
Significance of NLRP3 inflammasome in leukemias. Oxidative stress and mitochondrial dysfunction in leukemia cells could activate NLRP3 inflammasomes through mitochondrial ROS, potassium efflux, and NF-κB signaling pathways. NLRP3 activation leads to the generation of active IL-1β and IL-18 cytokines, and could cause pyroptosis. Further, NLRP3 activation plays various roles in different leukemias. For example, in Acute Myeloid Leukemia (AML), NLRP3 promotes immune evasion and survival, while inhibition reduces the disease burden. In Chronic Myeloid Leukemia (CML), NLRP3 is linked to KRAS mutations and therapy resistance. In Acute Lymphoblastic Leukemia (ALL), NLRP3 activation is correlated with glucocorticoid resistance, and in Chronic Lymphocytic Leukemia (CLL), P2X7R overexpression leads to increased NLRP3.

Very few studies have suggested that there is also a role for NLRP3 in CLL progression. Adinolfi et al. [70] have investigated P2X purinoceptor 7 (P2X7R) expression and its function in 21 patients with B-cell CLL. They found that lymphocytes from those with the evolutive variant had higher resting cytoplasmic Ca++ concentration and more significant Ca++ influx upon P2X7R stimulation, correlating with increased P2X7R expression. Further, they have shown that extracellular ATP reduced the spontaneous proliferation of lymphocytes in patients with the evolutive variant, but did not affect those with the indolent form. This study suggests an association between P2X7R expression and function in the progression of CLL. Similarly, Salaro et al. [71] have also shown overexpression of P2X7R and inhibition of NLRP3 in the lymphocytes of 23 CLL patients. Thus, recent studies suggest that the NLRP3 inflammasome-mediated immune and inflammatory responses play a significant role in leukemia, contributing to disease progression, drug resistance, and therapy response (Figure 3). These studies indicated that, depending upon the type of leukemia and resistance to therapy, NLRP3 inflammasome could be targeted as a potential therapeutic approach.

## 4. NLRP3 in Breast Cancer

Breast cancer is the most diagnosed cancer globally. The American Cancer Society estimated that in 2024, approximately 310,720 new cases of invasive breast cancer will be diagnosed in women in the United States, along with 56,500 new cases of non-invasive breast cancer. Further, breast cancer is still the most common cancer in American women, accounting for approximately 30% of all new female cancer diagnoses each year. While the five-year relative survival rate for localized breast cancer is 99%, the overall number of breast cancer-related deaths in 2024 is projected to be 42,250 for women and 530 for men (https://www.cancer.org/cancer/types/breast-cancer (accessed on 30 August 2024)). Further, inflammatory breast cancer is a lethal form of breast cancer rarely seen in young women under age 40. Pathological inflammation, edema, and breast redness are associated with it. It can progress rapidly and metastasize in various organs.

The NLRP3 inflammasome-mediated inflammatory response plays a significant role in breast cancer progression and metastasis. Recent studies have shown the significance of NLRP3 inflammasome and its involvement in breast cancer progression and metastasis, indicating its potential as an innovative therapeutic target [72,73,74]. NLRP3 inflammasome activation has been associated with promoting breast tumor growth, progression, and aggressiveness, as well as with the pyroptotic death of cancer cells [72,73,74]. Additionally, inhibiting the NLRP3 inflammasome has been shown to suppress breast cancer cell growth and enhance anticancer immunity [75,76]. Innate immune cytokines such as IL-1β and IL-18 have been shown to promote breast cancer progression in animal models [77,78]. They have also been implicated in promoting the migration and invasion of breast cancer cells and induction of IL-1β secretion promote these cells’ epithelial–mesenchymal transition (EMT) [79]. Further, Reed et al. [80] have shown that inhibition of IL-1β prevents iFGFR1-induced early-stage mammary lesions. Zhou et al. [81] have shown that IL-1β is responsible for the increased invasiveness of breast cancer cells, leading to bone metastasis. Zerumbone is an orally active natural cyclic sesquiterpene isolated from *Zingiber zerumbet*. It exerts anti-proliferative, anti-inflammatory, anti-cancer, anti-bacterial and anti-mutagenic activities. Jeon et al. [82] have demonstrated that zerumbone prevents IL-1β-induced triple-negative breast cancer cell invasion. On the other hand, zerumbone has been shown to prevent IL-1β-induced invasion of triple-negative breast cancer cells by inhibiting the expression of IL-8 and matrix metallopeptidase 3 (MMP3) [83]. Further, IL-1β has been shown to promote tumor invasiveness in breast cancer by activating the IL-1β/IL-1RI/β-catenin signaling pathway, leading to β-catenin accumulation and nuclear translocation [84]. Garcia-Morales et al. [85] have also shown that cannabidiol (CBD) prevents breast cancer cell viability, blocks the IL-1β/IL-1RI/β-catenin signaling pathway, restores epithelial organization, and prevents nuclear translocation of β-catenin, ultimately indicating a potential therapeutic role for CBD in counteracting the malignant features induced by IL-1β. Another study by Pham et al. [86] has shown the role of adiponectin, particularly its globular form (gAcrp), in suppressing breast cancer cell growth by inhibiting inflammasome activation. The study demonstrates that gAcrp significantly inhibits the activation of NLRP3 inflammasome components. This inhibition blunts the inflammatory response, leading to decreased cell viability, increased apoptosis, and causing cell cycle arrest via sestrin2 (SESN2) induction and adenosine monophosphate-activated protein kinase (AMPK) activation-dependent mechanisms. Similarly, Chen et al. [87] have shown that breast cancer susceptibility gene BRCA1 deficiency disrupts mitochondrial dynamics, leading to impaired stress-induced mitophagy and the activation of the NLRP3 inflammasome, which promotes a tumor-associated microenvironment and facilitates cancer progression. This study, thus, suggests that inhibiting inflammasome activation could be a potential therapeutic strategy to control tumor recurrence and metastasis in BRCA1-related breast cancer.

**Figure 4:**
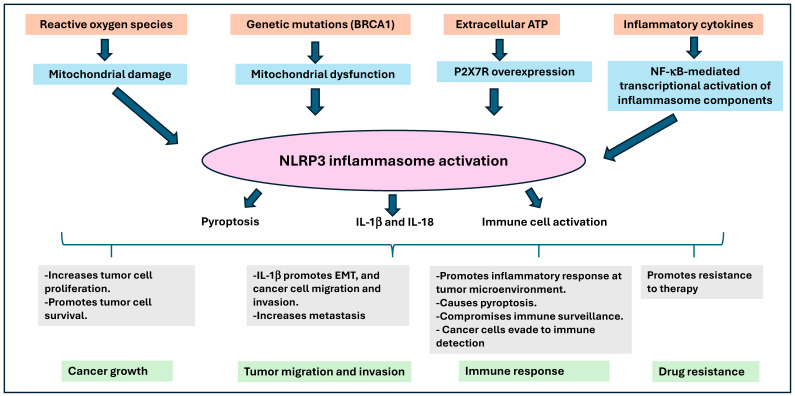
Role of NLRP3 inflammasome in breast cancer growth and spread. Several factors such as reactive oxygen species (ROS)-induced mitochondrial damage, BRCA1-associated genetic mutations causing mitochondrial dysfunction, extracellular ATP leading to P2X7R overexpression, and inflammatory cytokines that activate NF-κB-mediated inflammasome components could lead to activation of NLRP3 inflammasome. NLRP3-mediated release of IL-1β and IL-18 promotes cancer cell proliferation, survival, migration, immune evasion, and resistance to therapy. Further, the outcomes also include increased tumor growth, metastasis, compromised immune surveillance, and drug resistance.

Further, NLRP3 inflammasome inactivation driven by microRNA (miR)-223-3p has been shown to reduce tumor growth and increase anticancer immunity in breast cancer [88]. Xia et al. [89] have shown that P2X7 receptor activation promotes breast cancer cell invasion and migration by activating protein kinase B (AKT) signaling. Moreover, blocking P2X7 has demonstrated inhibition of breast cancer growth in mice through the NLRP3/caspase-1 pathway [90,91]. Thus, previous studies have suggested that the NLRP3 inflammasome exerts a significant influence on breast cancer progression, metastasis, and immune response. Modulating NLRP3 inflammasome activity has been proposed as a potential therapeutic strategy for breast cancer treatment (Figure 4). However, the precise mechanisms involved in NLRP3 inflammasome activation and its specific impact on the pathophysiology of breast cancer development need further investigations to demonstrate its potential use as a therapeutic target.

## 5. NLRP3 in Lung Cancer

Worldwide, more than 1 million people die due to lung cancer each year. It is estimated that cigarette smoking is the main reason for almost 90% of lung cancer risk in men and 70 to 80% in women [92]. Lung cancer is the second most common cancer and the cause of the most cancer-related deaths in both men and women in the U.S. [93]. The American Cancer Society estimated that in 2024, ~234,580 new cases and ~125,070 deaths will occur from lung cancer, non-small-cell and small-cell combined, in the U.S. The transition of normal lung epithelium from adenoma to carcinoma is associated with a variety of molecular and biochemical events, such as genetic alterations, epithelial cell proliferation/differentiation, and inflammation [94,95]. Primary initiators of carcinogenesis include (a) cells that suffered irreparable DNA damage due to increased free radicals, which cause activation of specific nucleases and damage DNA, RNA, proteins, and lipids; (b) loss of extracellular stimulation that regulates cell growth; (c) upregulation of growth factors and their receptors, and (d) autosomal dominant inheritance of cancer genes in multiple family members [94,95]. Dietary and environmental factors also play an essential role in predisposition to carcinogenesis. Furthermore, chronic inflammatory diseases such as chronic obstructive pulmonary disease (COPD), and infectious diseases such as tuberculosis are associated with an elevated risk of lung cancer [96,97,98,99]. Although it is not clear how cancer is initiated in the setting of chronic inflammation, increasing evidence strongly supports the association between lung cancer and inflammation [100,101].

Inflammation-mediated lung cancer is mainly associated with lung epithelial cells and alveolar macrophages. Several studies have shown NLRP3 inflammasome activation in these cells upon oxidant exposure. For example, cigarette smoke chemicals, including nicotine, have been shown to induce NLRP3 activation in human alveolar and bronchial epithelial cells [102,103,104]. Cigarette smoke-induced increase in oxidative stress and mitochondrial dysfunction could be responsible for the increase in NLRP3 activation, leading to lung carcinogenesis [105]. Further, increased expression of NLRP3 has been seen in mouse models of COPD [106]. Similarly, Rao et al. [107] have shown that in a mouse model of radiation-induced lung injury, there is a significant increase in the activation of NLRP3 inflammasome. Li et al. [108] have also shown that targeting NLRP3 inflammasome could enhance potential radiotherapy in non-small-cell lung cancer.

On the other hand, Zhang et al. [109] have indicated that cigarette smoke prevents the inflammasome complex formation and release of IL-1β in mice alveolar macrophages. Additionally, long-term cigarette smoke has been shown to cause NLRP3 activation in the oral mucosal epithelium [110]. Since oxidative stress and chronic inflammation are common in both COPD and lung cancer, the risk of developing lung cancer in COPD is always higher. Although epigenetic alterations and genetic mutations are major causes of lung cancer, COPD patients have an increased risk of developing lung cancer. However, several studies have supported the role of NLRP3 in lung cancer. A study by Wang et al. [111] has shown that increased NLRP3 activation is responsible for the growth and migration of lung cancer cells.

**Figure 5:**
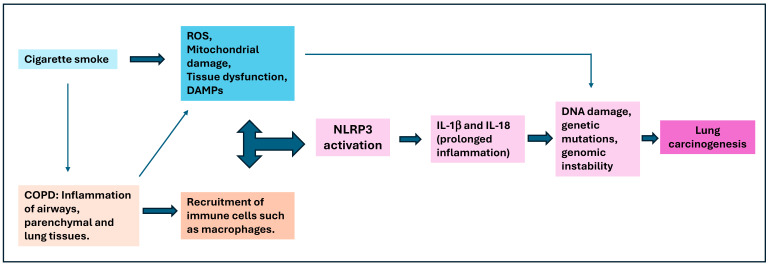
Significance of NLRP3 inflammasome activation in cigarette smoke and COPD-induced lung cancer development. Cigarette smoke leads to reactive oxygen species (ROS), mitochondrial damage, and tissue dysfunction, activating the NLRP3 inflammasome. The release of pro-inflammatory cytokines IL-1β and IL-18 contributes to prolonged inflammation. COPD-induced inflammation and immune cell recruitment, such as macrophages, further amplify this process. The persistent inflammation and oxidative stress promote DNA damage, genetic mutations, and genomic instability, ultimately leading to lung carcinogenesis.

Another study by Dutkowska et al. [112] has shown increased expression of IL-1β in non-small-cell lung cancer tumor tissues. Similarly, Kong et al. [113] have shown increased NLRP3 inflammasome in lung adenocarcinoma and small-cell lung cancer. The significance of NLRP3 in lung cancer is further confirmed by a mouse study where NLRP3-null mice inhibit lung cancer growth induced by benzo(a)pyrene without or with lipopolysaccharide (LPS) cotreatment [114]. Further, inflammasome-released cytokine IL-1β has been shown to promote metastasis of lung cancer cells through adhesion, invasion, and angiogenesis processes [115]. Similarly, Li et al. [116] have shown that NLRP3-mediated release of IL-1β and IL-18 increases the proliferation and migration of non-small-cell lung cancer cells. Recently, Metwally et al. have identified the fact that IL-1β rs16944 variants are associated with the development of non-small-cell lung cancer [117]. Furthermore, NLRP3 activation has been associated with promoting the proliferation and migration of lung cancer cells, impacting cancer cell viability and survival [118]. Conversely, some studies suggest a protective role of NLRP3 in lung cancer. NLRP3 expression is downregulated in non-small-cell lung cancers, with its expression positively correlating with overall survival [119]. Several studies indicate that lung cancer chemopreventive effects of some of the antioxidants are mediated by inhibiting NLRP3 inflammasome. For example, the anticancer effects of plant products reniformin A, saikosponin-D, and EEBR are exerted by inducing NLRP3-mediated pyroptosis in non-small-cell lung cancer cells [120,121,122]. Similarly, Zhao et al. [123] have shown that by inhibiting NLRP3, melatonin could regulate angiogenesis and lymphangiogenesis in lung adenocarcinoma. The role of NLRP3 in lung cancer is not clearly known; further studies are needed to elucidate the mechanisms underlying NLRP3 inflammasome’s beneficial and harmful impacts on lung cancer pathophysiology (Figure 5).

## 6. NLRP3 in Colon Cancer

Colon cancer is the third most common cancer and the second leading cause of cancer deaths in the world [124]. According to the National Cancer Institute, it is estimated that around 152,810 new cases of colon cancer and 53,010 deaths could happen due to this cancer (https://seer.cancer.gov/statfacts/html/colorect.html (accessed on 9 May 2024)). The most common cause of colon cancer is due to genetic predispositions, such as familial adenomatous polyposis and hereditary non-polyposis colon cancer (Lynch syndrome) [125,126]. Further, inflammatory bowel diseases (IBDs) such as ulcerative colitis and Crohn’s disease, poor lifestyle, and increased consumption of processed meat and alcohol are some of the major risk factors for developing colon cancer [127]. The risk of colon cancer has been shown to increase with age. Individuals fifty years and older have been shown to have an increased risk of colon cancer [124]. Increased oxidative stress and inflammatory response have also been associated with the elevated risk of colon cancer [128,129]. IBD is an inflammatory complication, and most of the cases of colon cancer are also associated with it. Further, recent studies using various antioxidants have also suggested that oxidative stress-regulated and NLRP3 inflammasome-mediated innate immune and inflammatory response could also contribute to colon cancer growth and spread [130].

In IBD, the activation of NLRP3 inflammasome-mediated IL-1β release has been shown to be a major risk factor for developing colon cancer [131]. NLRP3 inflammasome has a potential impact on colon carcinogenesis, inflammation, and possible therapeutic interventions, including chemo- and immuno-therapies. Further, in IBD patients, NLRP3 activation has been shown in the intestinal epithelial cells, and polymorphisms in IL-1β and 1L-18 have been associated with ulcerative colitis patients. Indeed, increased IL-1β has been demonstrated in patients with IBD [132]. Further, IL-1β- and NLRP3-null mice have shown significantly reduced inflammatory response and colitis development [133,134]. Administration of IL-1β to NLRP3-null mice has shown protection against oxazolone-induced colitis [135]. Further, small molecular inhibitors such as andrographolide have been shown to protect against azoxymethane and dextran sodium sulfate-induced colon cancer by inhibiting the NLRP3 inflammasome-mediated inflammatory response [136]. Further, several plant-based antioxidants and synthetic small molecular inhibitors have been shown to prevent IBD by inhibiting the NLRP3 inflammasome and expression of inflammatory markers such as IL-1β, IL-6, INF-γ, and IL-2 [130,137]. Hu et al. [138] have also shown that drugs that prevent the palmitoylation of NLRP3 could reduce the symptoms associated with dextran sodium sulfate-induced colitis in a mouse model. Further, in an acetic acid-induced colitis model and caco-2 colon epithelial cells, cyclooxygenase-2 (COX-2) inhibitor (mangiferin) prevents activation of NLRP3 and expression of inflammatory markers such as IL-1β, TNF-α, IL-16, and IFN-γ [139]. Similarly, quercetin has been shown to prevent NLRP3 inflammasome in HCT116 and HT29 colon cancer cells and a nude-mouse xenograft model [140].

Further, Allen et al. have shown that NLRP3 inflammasome has been suggested to function as a negative regulator of tumorigenesis during colitis-associated cancer (CAC) [141]. Similarly, activation of inflammasome-mediated caspase-1 by promoting cancer cell apoptosis could prevent colon cancer [142]. Knockdown or inhibition of NLRP3 activation has been shown to increase colonic inflammation, indicating a potential therapeutic avenue for managing colitis-associated cancer [143]. Similarly, it has been shown that Zinc finger protein 70 increases the IL-1β secretion in macrophages, promoting colon cancer cell proliferation (HCT-116). They have also demonstrated that the knockdown of zinc finger protein 70 could inhibit NLRP3 and azoxymethane/dextran sulfate sodium (AOM/DSS)-induced colon cancer [144]. In addition, gut microbiota plays an essential role in controlling inflammation. A few studies also indicate that NLRP3 inhibition prevents IBD by regulating the gut microbiota [145]. Similarly, Gao et al. [146], using a DSS-induced colitis model, have also demonstrated that by improving the gut microbiota, an herbal Chinese medicine could prevent NLRP3-mediated expression of inflammatory markers. They have also shown that NLRP3 is also expressed in both immune cells and colonic epithelial cells, suggesting a possible association with colon cancer. The NLRP3 inflammasome has been associated with the regulation of intestinal homeostasis, with its activation linked to inflammation-induced cancer [147].

**Figure 6:**
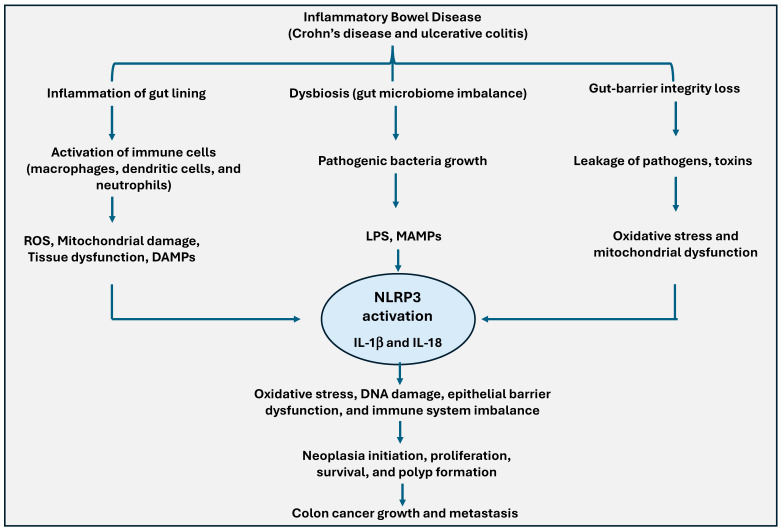
Role of NLRP3 inflammasome activation in promoting colon cancer development. During inflammatory bowel disease (IBD), such as Crohn’s disease and ulcerative colitis, the inflammation of the gut lining activates immune cells, causing oxidative stress, mitochondrial damage, and the release of DAMPs. Gut microbiome imbalance (dysbiosis) leads to pathogenic bacterial growth and loss of gut-barrier integrity, allowing pathogen and toxin leakage, further driving oxidative stress. These pathways cause NLRP3 activation and trigger IL-1β and IL-18 release. Increased inflammasome response results in immune system imbalance, epithelial-barrier dysfunction, and neoplasia initiation, ultimately contributing to colon cancer growth and metastasis.

Furthermore, the NLRP3 inflammasome has been implicated in the proliferation, migration, and invasion of cancer cells in gastrointestinal cancers, including colon cancer [148]. NLRP3 inhibition has also been shown to prevent the growth and migration of non-small-cell lung cancer cells, indicating a broader role in promoting cancer progression [149]. Additionally, Zhang et al. have shown that NLRP3 inflammasome is involved in the spread of colon cancer by increasing the epithelial–mesenchymal transition (EMT), a process essential for migration and invasion [150]. They have shown that NLRP3 activation and release of IL-1β mediates macrophage-mediated increase in the invasion and migration of colon cancer cells through regulating the EMT (Figure 6). Similarly, Deng et al. [151] have also shown that NLRP3 activation in macrophages mediates colon cancer migration and invasion. So far, these studies suggest that NLRP3 inflammasome mediates colon cancer migration and invasion.

## 7. NLRP3 in Gastric and Pancreatic Cancers

Gastric cancer is the fifth most common cancer worldwide [152]. Most gastric cancers are associated with the *Helicobactor pylori* (*H. pylori*) infections [152,153]. Moreover, genetic mutations in the CDH1 gene (familial hereditary diffuse gastric cancer), alcohol consumption, processed meats, and high salt preserved foods could also contribute to the risk of developing gastric cancer [154]. Inflammation is a key in the development of gastric cancer, especially during *H. pylori* infections. The bacteria are known to colonize the lining of the stomach and induce prolonged inflammation, leading to chronic gastritis. Thus, chronic inflammation could lead to damage to the gastric epithelial cells, causing intestinal metaplasia and gastric adenocarcinoma [155]. NLRP3-mediated immune response has also been associated with chronic gastritis. Specifically, a few studies have shown that *H. pylori* activates NLRP3 and releases inflammatory IL-1β and IL-18 cytokines [156,157].

Further, several studies have shown that NLRP3 is highly expressed in stomach adenocarcinoma, and could serve as a potential prognostic biomarker for gastric cancer [148,158,159]. *H. pylori* infection has been associated with enhanced ROS production and NLRP3 expression in gastric cancer, triggering alterations in the M1 and M2 macrophage polarization [160]. Silencing NLRP3 has been demonstrated to reduce the effects of CagA on gastric cancer-cell migration and invasion, suggesting a potential role of NLRP3 in promoting the metastatic potential of gastric cancer [161]. Additionally, the NLRP3 inflammasome has been linked to the regulation of IL-1β production, which is implicated in gastric cancer development [162,163]. IL-18 has also been shown to contribute to increased inflammatory response in a DSS-induced IBD model [164]. Further, Chen et al. [165] have also shown that *H. pylori* causes mitochondrial oxidative damage and dysfunction, helping bacteria evade the host’s immune response. They have shown that by increasing mitophagy, *H. pylori* prevents NLRP3 inflammasome, leading to the development of gastric cancer. Similarly, Qi et al. [166] have shown that flavonoids such as 3.4.5.7-tetrahydroxyflavone induce mitochondrial damage, which leads to activation of NF-κB/NLRP3-induced pyroptosis in human gastric adenocarcinoma cells. Liu et al. [167] also indicate that Baicalin promotes gastric cell pyroptosis by activating NF-κB and NLRP3 signaling pathways. Further, bile acids have been shown to cause mitochondrial tethering to mitochondria, which can cause increased calcium overload and NLRP3 activation [168,169].

Further, paclitaxel has been shown to prevent NLRP3-mediated migration and invasion of gastric cells, suggesting NLRP3’s involvement in the metastasis of gastric cancer [170]. Furthermore, overexpressed long non-coding RNA ADAMTS9-AS2 has been shown to inhibit gastric cancer development by regulating the miR-223-3p/NLRP3 axis, suggesting a potential tumor-suppressive role for NLRP3 in gastric cancer [171]. A few studies have also indicated that gut microbiota has been shown to be involved in gastric cancer progression (Table 1). For example, *H. pylori* infection has been shown to disrupt gut microbial homeostasis, leading to increased NLRP3-mediated inflammatory response [172,173]. Thus, these studies suggest that NLRP3 plays a critical role in *H. pylori*-induced gastric cancer.

## 8. NLRP3 in Pancreatic Cancer

Pancreatic cancer, or pancreatic ductal carcinoma, is rare, and accounts for 3% of all cancers. According to the American Cancer Society in the U.S., in 2024, an estimated 66,440 people may be diagnosed with pancreatic cancer, and around 51,750 people may die from it (https://www.cancer.org/cancer/types/pancreatic-cancer/about/key-statistics.html (accessed on 9 December 2024)). Although genetic mutations, obesity, and smoking are major risk factors for the development of pancreatic cancer, inflammation of the pancreas, pancreatitis, is one of the major causes [174].

Oxidative stress, as well as inflammatory responses mediated by the NF-κB and NLRP3 pathways, have been shown to induce pancreatitis [175,176,177]. Specifically, pro-inflammatory cytokines expressed by NF-κB, such as TNF-α, IL-6, IFN-γ, and IL-8, and innate immune cytokines released by NLRP3, such as IL-1β and IL-18, play a significant role in pancreatitis. Xu et al. [178] have shown that in pancreatic acinar cells, IL-1β is involved in the formation of trypsin and inhibition of cell growth. Further, Caronni et al. [179] have suggested the importance of tumor-associated macrophage-mediated inflammatory responses in pancreatic cancer. They have demonstrated that pancreatic cancer progression can be inhibited by preventing the interactions between prostaglandin E2 (PGE2) and IL-1β in the tumor-associated macrophages. Previously, a study by Chen et al. [180] has also shown that pancreatic ductal adenocarcinoma cell debris activates NF-κB signaling in the M2 macrophages and releases IL-1β. They have also demonstrated that IL-1β is responsible for EMT and metastasis of pancreatic cancer. Further, Wu et al. [181] have also demonstrated that HMGB1 mediates the NLRP3-mediated processing of IL-1β in macrophages and pancreatic cells, leading to pancreatic injury.

In addition, several studies have noted the influence of NLRP3 in pancreatic cancer (Table 1). For instance, NLRP3 has been associated with increased frequency of the rs35829419-NLRP3 polymorphism in patients with pancreatic cancer, suggesting a potential genetic association with the disease [182]. Additionally, upregulation of NLRP3 signaling has been linked to promoting platelet activation and aggregation, leading to tumor growth and metastasis in a mouse model of pancreatic cancer [183]. These studies suggest that NLRP3 inflammasome has been associated with an increased risk of inflammation-induced pancreatic cancer.

In addition, some studies have suggested the protective role of NLRP3 in pancreatic cancer. For example, downregulation of NLRP3 has been shown to inhibit the proliferation and migration of pancreatic cancer cells, suggesting a potential tumor-suppressive role [184]. Moreover, the NLRP3 inflammasome has been implicated in promoting pancreatic islet damage, indicating its possible involvement in the pathophysiology of pancreatic cancer [185]. The studies on the prevention of pancreatitis further support the role of NLRP3 by using various antioxidants in animal models. For example, Ren et al. [186] have shown that the natural plant product Danshensu prevents the activation of NF-κB and NLRP3 in an acute-pancreatitis mouse model. Similarly, pinocembrin, pramipexole, proanthocyanidins, and Emodin inhibit NLRP3-mediated caspase-1-1 activation and IL-1β production in animal models of acute pancreatitis [187,188,189,190]. These studies indicate the beneficial and harmful effects of NLRP3 in the development of pancreatic cancer. However, additional studies are still needed to clarify the role of NLRP3-mediated immune and inflammatory responses in pancreatic cancer progression and therapy.

## 9. NLRP3 in Prostate Cancer

Prostate cancer is one of the most common cancers in men, and it is estimated that in 2024, around 299,010 new cases of prostate cancer and 35,250 deaths might happen from it (https://www.cancer.org/cancer/types/prostate-cancer/about/key-statistics.html (accessed on 9 December 2024). Recent studies also suggest that systemic inflammation also plays a major role in prostate tumor progression and metastasis [191,192]. When compared to other cancers, the role of NLRP3 in prostate cancer is not well investigated. Although few studies indicate the beneficial role of NLRP3 inflammasome inhibition in prostate cancer using animal models, clinical studies are required to understand its role in the progression of prostate cancer. Expression of inflammasome proteins has been observed in the human prostate epithelial cells [193]. Further, expression of NLRP3 in prostate cancer cells (PC-3) has been shown to be increased under hypoxia [194]. Xu et al. [195] have also shown the expression of NLRP3 in LNCaP and PC3 cells and prostate cancer tissues. Further, they have shown that activation of NLRP3 mediates prostate cancer cell proliferation and migration, and inhibition of NLRP3 prevents it. Similarly, pyroptosis mediated by caspase-1 has been shown to be involved in prostate cancer progression [196]. Moreover, the NLRP3 inflammasome has been implicated in promoting prostate islet damage, indicating its potential involvement in the pathophysiology of prostate cancer [197]. The involvement of NLRP3 in prostate cancer is further supported by a study from Zeng et al. [198]. They have shown that Carvedilol (CVL), a β-adrenergic receptor antagonist, causes NLRP3 inflammasome-mediated pyroptosis in prostate cancer.

Furthermore, the upregulation of NLRP3 signaling has been linked to the development of prostate inflammatory lesions in rat models of pancreatitis when treated with a combination of estrogen benzoate and a high-fat diet [199]. Chronic pancreatic atrophy and inflammation are associated with the development of prostate cancer. Similarly, NLRP3 has been associated with tumor–node–metastasis staging in prostate cancer [200]. Another study by Chaudagar et al. [201] suggested that NLRP3 is highly expressed in tumor-associated macrophages in patients undergoing androgen-deprivation treatment. Here, androgen receptor inhibition has been shown to increase NLRP3 expression, but not activity. Further, they have shown that a combination of androgen-deprivation treatment. along with inhibition of NLRP3. prevents prostate cancer in a mouse model. Similarly, NLRP3 acetylation and inhibition of inflammasome complex formation by androgen receptor inhibitor could block prostate cancer progression in nude-mice xenografts [202]. A recent study by Liu et al. [203] has shown that ulinastatin, a protease inhibitor, prevents prostate cancer in a Rho/Rock/NLRP3 inflammasome pathway in PC-3 cells. In addition, LPS + Nigericin-induced NLRP3 inflammasome has been shown to be inhibited by doxycycline [204]. Similarly, Natriuretic peptides such as Atrial Natriuretic Peptides (ANPs) and B-Type Natriuretic Peptide (BNP) have been found to inhibit NLRP3 inflammasome and its associated inflammatory response in prostate cancer [205]. Thus, a few studies indicate that activation of the NLRP3 inflammasome has been associated with promoting inflammation-induced carcinogenesis in prostate cancer. However, additional studies are required to understand the molecular pathways through which inflammasome-mediated inflammatory response is involved under normal, hypoxia, androgen receptor-mediated progression and metastasis of prostate cancer.

## 10. NLRP3 in Gynecological Cancers

Gynecological cancers, including ovarian, cervical, and endometrial cancers, are significant health concerns, globally [206]. Uncontrolled growth of endometrial tissue outside of the uterine cavity causes endometriosis. Inflammation plays a major influence in the progression of endometriosis [207]. Specifically, alterations in the immune cell function, such as NK cell and T-cell functions, and the phenotype could lead to endometriosis [208]. Few studies have indicated that NLRP3 activation causes endometrial cell apoptosis. Further, NLRP3 activation has been associated with endometriosis, where defective macrophages and endometrial stromal cells elevate the expression of NLPR3 [209]. This study has demonstrated that IL-1β promotes endometrial cell migration and lesion development in a mouse model, and the inhibition of NLRP3 prevents lesion size and cell migration. Estrogen has been shown to activate NLRP3 inflammasome through the estrogen response element, and is involved in the pathology of endometriosis [210]. Further, Zhang et al. [211] have shown that in endometrial tissues, increased angiogenesis is associated with increased Notch1 signaling and NLRP3-induced pyroptosis. Similarly, Li et al. [212]. have shown that NLRP3 inflammasome activation through IncRNA NEAT/miR-141-3p/HTRA1 pathway leads to endometriosis. In addition, Hang et al. [213] have also shown that tripartite motif-containing 24 (TRIM24) is involved in the endometriosis progression through ubiquitination of NLRP3 and pyroptosis.

Further, a cross-sectional study by Fonseca et al. [214] has investigated NLRP3 inflammasome in follicular fluid and granulosa cells in women undergoing controlled ovarian stimulation. They found that IL-1β and IL-18 levels were elevated in the follicular fluid in the endometriosis group, compared to the non-endometriosis group. Similarly, Huang et al. [215] have found that the expression of PGE2 and NLRP3 inflammasome-related proteins, such as cleaved caspase-1, IL-1β, and IL-18, increased in the endometrial tissues. The significance of NLRP3 inflammasome in endometriosis is further confirmed by studies using various antioxidants and NLRP3 inhibitors. For example, fisetin has been shown to prevent oxidative stress, Poly ADP ribose expression, and to cause apoptosis in endometrial cells through the NLRP3 pathway [216]. Similarly, MCC950 (an NLRP3 inhibitor) prevents ovarian endometriosis and the expression of NLRP3 and IL-1β in the endometrial stromal cells and cyst-derived stromal cells. Alpha-lipoic acid has been shown to prevent endometriosis by preventing the NLRP3-mediated release of IL-1β and IL-18 [217].

Ovarian cancer is one of the major causes of cancer-related deaths in women because of late diagnosis and metastasis [218]. Although inflammation has been shown to be a risk factor responsible for developing ovarian cancer, the role of NLRP3 inflammasome in ovarian cancer is not well studied when compared to other types of cancers. However, a few studies have highlighted NLRP3’s role in tumorigenesis and cancer progression in ovarian cancers (Table 2) [219]. Initially, Chang et al. [220] have identified that NLRP3, IL-1β, and IL-18 genes have been upregulated during the transformation of endometriosis to ovarian cancer. Later, Luborsky et al. [221] found that inflammasome complex proteins and related cytokines are elevated in hen and human ovarian cancers. Further, Solini et al. [222] have shown the involvement of adipocyte P2X7R-NLRP3 inflammasome in regulating the chemotaxis and metastasis of epithelial ovarian cancer. It has been shown that increased NLRP3 in ovarian cancer is associated with overall survival [223]. Increased expression of NLRP3 inflammasome and activation of IL-1β has been associated with the progression of ovarian endometriosis, and inhibitor NLRP3 using MCC950 prevents ovarian endometriosis [224]. Similarly, citric acid has been shown to prevent ovarian cancer cell growth through caspase-4, thioredoxin-interacting protein, and NLRP3 inflammasome-mediated pyroptosis [225]. Further, a natural antioxidant, polydatin, has been shown to prevent ovarian and cervical cancers by inhibiting NLRP3 inflammasome activation. The significance of NLRP3 activation in ovarian cancer is further confirmed by a study by Li et al. [226], where they found that NLRP3 is overexpressed in ovarian cancer and is correlated with the poor survival rate and cisplatin resistance to ovarian cancer. This study also demonstrated that silencing of NLRP3 prevents EMT and sensitizes cisplatin chemotherapy. Similarly, another study by Wu et al. [227] has indicated that RAS-associated C3 botulinum toxin substrate 1 (RAC1) expression is associated with paclitaxel resistance to ovarian cancer. They have shown that RAC1 promotes paclitaxel resistance by inhibiting the PAK4/MAPK pathway, as well as caspase-1 and GSDMD-mediated pyroptosis.

Cervical cancer is one of the major cancer-related deaths in females globally. According to the American Cancer Society, in the USA for 2024, it is estimated that around 13,280 new cases of invasive cervical cancer and around 4360 cervical cancer-related deaths could be possible (https://www.cancer.org/cancer/types/cervical-cancer/about/key-statistics.html (accessed on 24 September 2024)). Inflammation is one of the major causes of the progression of cervical cancer. Specifically, viral infections such as human papillomavirus (HPV)-induced inflammation have been associated with the development of cervical cancer. Further, Pontillo et al. [228] have indicated that single-nucleotide polymorphisms in inflammasome genes such as NLRP3 and others could be associated with the HPV-induced progression of cervical cancer. They found that in case-control analysis, IL-1β rs1143643 is linked with HPV protection, while NLRP3 rs10754558 has been linked with a lower risk of infection with HPV. Further, infection with Chlamydia trachomatis has been shown to increase NLRP3-dependent caspase-1 activation in cervical endothelial cells [229]. Similarly, Lu et al. [230] have found that NLRP3 rs10754558 is associated with an increased risk of cervical cancer. The therapeutic benefit of inhibiting NLRP3 inflammasome is further confirmed by a study using the CD200R1 agonist, CD200Fc. This study found that CD200Fc inhibits LPS-induced NLRP3 inflammasome activation and release of IL-1β in SiHa and Caski human cervical cancer cells [231]. Further, Yu et al. [232] have conducted a clinical study using 50 cervical cancer patients and age-matched controls, and they found that miRNA-214 increases the NLRP3 and [232,233] causes pyroptosis, and thus prevents cervical cancer cell proliferation. Further, NLRP3 inflammasome activation has been linked to promoting pyroptosis, a form of programmed cell death, in cervical and ovarian cancer cells, affecting cancer cell viability and survival [232,233]. In addition, NLRP3 has been implicated in promoting epithelial–mesenchymal transition (EMT) in ovarian and cervical cancer cells, a critical process for cancer metastasis and progression [234]. A recent study by Fernandes et al. [235] has indicated that poor overall survival of cervical cancer is associated with overexpression of IL-1β. Further, Ji et al. [236] have demonstrated that the oncogene Foxm1 induces NLRP3 inflammasome activation and promotes CD8+ T-cell mediated immunosuppression in cervical cancer.

**Table 2:** Role of NLRP3 inflammasome in gynecological cancers.

Cancer Type	Role of NLRP3	Citations
Endometriosis	Elevated NLRP3 expression in defective macrophages and endometrial stromal cells is associated with endometriosis.	[209]
	IL-1β promotes endometrial cell migration and lesion development, and NLRP3 inhibition reduces lesion size.	[209]
	Estrogen activates NLRP3 through the estrogen response element, linking it to endometriosis pathology.	[210]
	NLRP3-induced pyroptosis is associated with increased Notch1 signaling and angiogenesis in endometrial tissues.	[211]
	NLRP3 inflammasome activation through IncRNA NEAT/miR-141-3p/HTRA1 pathway contributes to endometriosis.	[212]
	TRIM24, through NLRP3 ubiquitination and pyroptosis, causes endometriosis.	[213]
	Elevated IL-1β and IL-18 levels are seen in follicular fluid of endometriosis patients undergoing ovarian stimulation.	[214]
	PGE2- and NLRP3-related protein expressions are increased in endometrial tissues.	[215]
	Antioxidants like fisetin, MCC950, and alpha-lipoic acid inhibit NLRP3-mediated inflammation and prevent endometriosis.	[217]
Ovarian cancer	NLRP3, IL-1β, and IL-18 are upregulated during transformation of endometriosis to ovarian cancer.	[220]
	Elevated inflammasome complex proteins are seen in hen and human ovarian cancers.	[221]
	Adipocyte P2X7R-NLRP3 inflammasome shown to regulate chemotaxis and metastasis.	[222]
	High NLRP3 expression is linked to poor survival and cisplatin resistance, and silencing NLRP3 sensitizes chemotherapy.	[226]
	Polydatin inhibits NLRP3 inflammasome activation and prevents ovarian cancer progression.	[225]
	Citric acid prevents ovarian cancer cell growth via NLRP3-mediated pyroptosis.	[225]
	RAC1 expression linked to paclitaxel resistance through PAK4/MAPK pathway and NLRP3-mediated pyroptosis.	[227]
Cervical cancer	NLRP3 polymorphisms are linked to HPV infection risk and cervical cancer progression.	[228]
	Chlamydia trachomatis infection increases NLRP3-dependent caspase-1 activation.	[229]
	NLRP3 rs10754558 is associated with increased cervical cancer risk.	[230]
	CD200Fc inhibits NLRP3 activation in cervical cancer cells by reducing IL-1β production.	[231]
	miRNA-214 increases NLRP3, promotes pyroptosis and reduces cervical cancer cell proliferation.	[232]
	NLRP3-mediated EMT is implicated in cancer metastasis.	[234]
	Foxm1 induces NLRP3 activation by promoting immunosuppression.	[236]

## 11. NLRP3 in Head and Neck Cancer

Head and neck cancer, where most of the cases are of squamous-cell carcinoma of the epithelial lining of the oral, pharynx, and larynx, is the seventh most common cancer worldwide [237]. Although tobacco chewing, alcohol, environmental smoke, and viral infections are the major causes of head and neck cancer (HNSCC), recent studies also indicate that inflammation could also play a role [238]. Specifically, the NLRP3 inflammasome activation in squamous-cell carcinoma tissues has been associated with the progression of HNSCC [239]. Some studies indicate that the involvement of IL-1β in cancer progression and the inhibition of IL-1β could prevent it [240]. Chen et al. have shown that expression of NLRP3 is increased in HNSCC tissues while IL-1β is increased in the peripheral blood of the patients [241]. They have also shown that increased NLRP3 inflammasome is associated with HNSCC progression, and its inhibition leads to decreased accumulation of immunosuppressive cells and increased effector T-cells. In a separate study, Chen et al. [242] have also shown that increased NLRP3 in tumor-associated macrophages leads to poor prognosis and increased HNSCC growth. Increased expression of NLRP3 and increased serum IL-1β has been observed in oral squamous-cell carcinoma (OSCC) patients [243]. Thus, the prognostic value of inflammasomes in head and neck carcinoma has also been highlighted, indicating their relevance in predicting patient outcomes [242,243]. Moreover, NLRP3 has been implicated in promoting the proliferation and migration of esophageal squamous-cell carcinoma, suggesting its involvement in cancer progression [244].

Similarly, Feng et al. have shown that increased NLRP3 and IL-1β production is correlated with the 5-fluorouracil chemoresistance in OSCC, and NLRP3- and IL-1β-null mice showed decreased tumor incidence [245]. On the other hand, Yang et al. [246] have shown that bitter melon-derived extracellular vesicles increase 5-FU-induced therapeutic efficacy and reduce resistance to 5-FU in OSCC cells. Further, in OSCC, zymosan from candida albicans fungal cell wall has been shown to promote IL-1β production and proliferation of oral squamous-cell carcinoma cells [247]. Chow et al. [248] have demonstrated that calcium regulator CD38 prevents NLRP3-mediated pyroptosis in HNSCC cell lines. Xiao et al. [249] have also shown that IL-6 is important in OSCC progression by increasing the JAK2, STAT3, Sox4, and NLRP3 signaling pathways. The metastasis of OSCC has been shown to be prevented by BAY-117082, an NLRP3 inflammasome inhibitor, in a mouse orthotopic model [250]. Similarly, melatonin has been shown to prevent OSCC by regulating the MIF, NLRP3, and IL-1β pathway [251]. Antioxidant bacopa monnieri prevents NLRP3 inflammasome through mitophagy and oral cancer growth in a 4-nitroquinolin-1-oxide and arecoline-induced mouse model [252]. Further, Coenzyme Q0 prevents HNSCC growth in xenograft mice by inhibiting NLRP3 expression [253]. These studies suggest that the NLRP3 inflammasome plays a significant role in head and neck cancers, with evidence suggesting its involvement in tumorigenesis and patient prognosis. Further research is needed to fully understand the mechanisms underlying NLRP3 inflammasome activation and its impact on head-and-neck-cancer pathophysiology.

## 12. Conclusions and Future Perspectives

Recent studies indicate that NLRP3 inflammasome is critically involved in the various aspects of cancer progression and metastasis by either altering the immune response at the tumor microenvironment or inducing the proliferation or pyroptosis of cancer cells. Although some studies clearly indicate that inhibition of NLRP3 is an innovative approach to control cancer growth and spread, its potential as a therapeutic target and a prognostic marker across different cancers needs further investigation. Those studies will help to understand the significance of NLRP3-mediated immune and inflammatory responses in cancer progression, and also help to identify the significance of inflammasomes in advancing cancer immunotherapy.

The activation of the NLRP3 inflammasome plays a pivotal role in cancer progression and the immune response, in a dual way. In one way, NLRP3 activation, by releasing pro-inflammatory cytokines such as IL-1β and IL-18 could lead to chronic inflammation, promoting tumor growth and spread. In another way, it can boost anti-tumor immunity by promoting cell death and attracting immune cells to the tumor site. This binary function is of utmost importance for the potential development of NLRP3 inhibitors as part of cancer therapy, which could inhibit inflammation-linked tumor progression. However, not all cancer types respond to similar immunological responses. Therefore, additional studies are required to identify which specific forms of cancer could benefit from NLRP3 inhibitors. Understanding treatment approaches could reduce the chemo-, hormone- and immune-resistance of cancer cells, enhance therapeutic efficacy, and diminish the possible side effects. Further, developing targeted NLRP3 inhibitors and their combination with other treatments could offer better treatment options for advanced cancer treatments with improved patient outcomes.

Although the prospects of using NLRP3 inhibitors in cancer therapy are promising, special attention needs to be taken when treating different cancers, as inflammasome could play a dual role in both promoting and inhibiting cancer. In some cancers, where inflammation plays a significant role, NLRP3 inhibitors could be crucial by diminishing inflammation and slowing tumor growth and spread. In these circumstances, NLRP3 inhibitors could reduce the pro-inflammatory cytokines and their mediated inflammatory responses that promote tumor growth and metastatic spread. However, additional clinical studies are necessary to address the significance of NLRP3 inhibitors in this direction.

In more resistant and advanced cancers, the combination of NLRP3 inhibitors, chemo drugs, and immune checkpoint inhibitors could enhance overall treatment efficacy. Indeed, some studies have shown that the combination of NLRP3 inhibitors with checkpoint inhibitors could improve therapeutic efficacy by modifying the immune surveillance in the tumor microenvironment and tumor cells. Recent studies have also identified novel biomarkers for various cancers, and understanding how NLRP3 inhibition alters these biomarker responses could help develop better therapeutic approaches. Further, the use of next-generation sequencing and omics could help to identify better drugs to inhibit NLRP3 inflammasome, and could also identify possible prognosis markers for cancer.

In addition, the current understanding of how the existing NLRP3 inhibitors exert any side-effects is important. This will help to develop specific inhibitors with lower side-effects when treating cancer patients. Generally, the inflammasome-mediated immune and inflammatory responses contribute to immune surveillance, pathogen recognition, and cell death; inhibiting it might imbalance the immune responses and increase the infection risk or other adverse effects. Therefore, additional safety-profile studies will be needed to ensure better cancer therapy with the NLRP3 inhibitors to control any unintended side-effects. Developing such new inhibitors with greater specificity and fewer off-target effects could improve their therapeutic potential. Although the potential use of NLRP3 inflammasome inhibitors in cancer therapy has been developing for the last decade, the therapeutic approach should be directed to the inflammasome’s specific role in different cancer types. This individualized treatment of each cancer type should be adjusted to the specific role of inflammasome in cancer progression or inhibition. Thus, by understanding how the NLRP3 inflammasome either promotes or inhibits cancer growth in various tissues, we can develop more precise and effective treatments, using NLRP3 inhibitors alone or in combination with other immunotherapy agents.

Thus, recent studies suggest that inhibition of NLRP3 inflammasome-mediated immune and inflammatory responses provides innovative strategies to control cancer growth and metastasis. Since NLRP3 might be involved in the tumor progression as well as pyroptosis of cancer cells, ongoing research should further explore the effects of NLRP3 inhibition on specific cancer types and stages, to understand how these inhibitors alter carcinogenic processes at different levels. Additionally, pre-clinical studies using advanced technologies could help better prognosis and treatment strategies with NLRP3 inhibitors alone or in combination with other therapeutic approaches, especially immunotherapies.

## Figures and Tables

**Table 1:** Significance of NLRP3 inflammasome in gastric and pancreatic cancers.

Cancer Type	Role of NLRP3	Citations
Gastric cancer	*H. pylori* infection activates NLRP3 and releases IL-1β and IL-18, leading to chronic inflammation.	[156,157]
	High NLRP3 expression observed in gastric adenocarcinoma serves as a potential prognostic biomarker.	[148,158,159]
	*H. pylori*-generated ROS enhances NLRP3 expression and alters macrophage polarization.	[160]
	Inhibition of NLRP3 reduces CagA-mediated gastric cancer cell migration.	[161]
	IL-1β regulation by NLRP3 linked to gastric cancer development.	[162,163]
	Mitochondrial oxidative damage from *H. pylori* influences NLRP3 activity.	[165,166,167]
	Paclitaxel prevents NLRP3-mediated migration and invasion.	[170]
	Long non-coding RNA ADAMTS9-AS2 inhibits gastric cancer through the miR-223-3p/NLRP3 axis.	[171]
	Gut microbiota disruptions by *H. pylori* enhance NLRP3-mediated inflammatory response.	[172,173]
Pancreatic Cancer	NLRP3 pathway activation induces pancreatitis and chronic inflammation.	[174,175,176,177]
	IL-1β promotes pancreatic injury and metastasis.	[178,179,180,181]
	Genetic polymorphisms in NLRP3 are linked to increased pancreatic cancer risk.	[182]
	NLRP3 promotes platelet activation and aggregation, which contributes to tumor growth.	[183]
	Downregulation of NLRP3 inhibits cancer cell proliferation, suggesting a tumor-suppressive role.	[184]
	NLRP3 is linked to islet damage and inflammation.	[185,186,187,188]

## References

[1] Shadab A., Mahjoor M., Abbasi-Kolli M., Afkhami H., Moeinian P., Safdarian A.R. (2023). Divergent functions of NLRP3 inflammasomes in cancer: A review. Cell Commun. Signal..

[2] Xu Z., Kombe Kombe A.J., Deng S., Zhang H., Wu S., Ruan J., Zhou Y., Jin T. (2024). NLRP inflammasomes in health and disease. Mol. Biomed..

[3] Stergiou I.E., Tsironis C., Papadakos S.P., Tsitsilonis O.E., Dimopoulos M.A., Theocharis S. (2024). Unraveling the role of the NLRP3 inflammasome in lymphoma: Implications in pathogenesis and therapeutic strategies. Int. J. Mol. Sci..

[4] Gouravani M., Khalili N., Razi S., Keshavarz-Fathi M., Khalili N., Rezaei N. (2020). The NLRP3 inflammasome: A therapeutic target for inflammation-associated cancers. Expert Rev. Clin. Immunol..

[5] Kelley N., Jeltema D., Duan Y., He Y. (2019). The NLRP3 inflammasome: An overview of mechanisms of activation and regulation. Int. J. Mol. Sci..

[6] Blevins H.M., Xu Y., Biby S., Zhang S. (2022). The NLRP3 inflammasome pathway: A review of mechanisms and inhibitors for the treatment of inflammatory diseases. Front. Aging Neurosci..

[7] Hamarsheh S., Zeiser R. (2020). NLRP3 inflammasome activation in cancer: A double-edged sword. Front. Immunol..

[8] Abderrazak A., Syrovets T., Couchie D., El Hadri K., Friguet B., Simmet T., Rouis M. (2015). NLRP3 inflammasome: From a danger signal sensor to a regulatory node of oxidative stress and inflammatory diseases. Redox Biol..

[9] Leemans J.C., Cassel S.L., Sutterwala F.S. (2011). Sensing damage by the NLRP3 inflammasome. Immunol. Rev..

[10] Shi F., Wei B., Lan T., Xiao Y., Quan X., Chen J., Zhao C., Gao J. (2021). Low NLRP3 expression predicts a better prognosis of colorectal cancer. Biosci. Rep..

[11] Zhao H., Xu J., Zhong Y., He S., Hao Z., Zhang B., Liu Z., Zhou X. (2024). Mammary hydroxylated oestrogen activates the NLRP3 inflammasome in tumor-associated macrophages to promote breast cancer progression and metastasis. Int. Immunopharmacol..

[12] Wang Y., Zhang H., Xu Y., Peng T., Meng X., Zou F. (2021). NLRP3 induces the autocrine secretion of IL-1β to promote epithelial-mesenchymal transition and metastasis in breast cancer. Biochem. Biophys. Res. Commun..

[13] Marandi Y., Hashemzade S., Tayebinia H., Karimi J., Zamani A., Khodadadi I. (2021). NLRP3-inflammasome activation is associated with epithelial-mesenchymal transition and progression of colorectal cancer. Iran. J. Basic. Med. Sci..

[14] Di Y., Wang Z., Xiao J., Zhang X., Ye L., Wen X., Qin J., Lu L., Wang X., He W. (2024). ACSL6-activated IL-18R1-NF-κB promotes IL-18-mediated tumor immune evasion and tumor progression. Sci. Adv..

[15] Wu D., Wei C., Li Y., Yang X., Zhou S. (2021). Pyroptosis, a new breakthrough in cancer treatment. Front. Oncol..

[16] Lin T.Y., Tsai M.C., Tu W., Yeh H.C., Wang S.C., Huang S.P., Li C.Y. (2021). Role of the NLRP3 inflammasome: Insights into cancer hallmarks. Front. Immunol..

[17] Moossavi M., Parsamanesh N., Bahrami A., Atkin S.L., Sahebkar A. (2018). Role of the NLRP3 inflammasome in cancer. Mol. Cancer..

[18] Sun Y., Shen Y., Liu Q., Zhang H., Jia L., Chai Y., Jiang H., Wu M., Li Y. (2024). Global trends in melanoma burden: A comprehensive analysis from the Global Burden of Disease Study, 1990–2021. J. Am. Acad. Dermatol..

[19] Colombino M., Casula M., Paliogiannis P., Manca A., Sini M.C., Pisano M., Santeufemia D.A., Cossu A., Palmieri G. (2024). Heterogeneous pathogenesis of melanoma: BRAF mutations and beyond. Crit. Rev. Oncol. Hematol..

[20] Shebrain A., Idris O.A., Jawad A., Zhang T., Xing Y. (2024). Advancements and challenges in personalized therapy for BRAF-mutant melanoma: A comprehensive review. J. Clin. Med..

[21] Mirek J., Bal W., Olbryt M. (2024). Melanoma genomics—Will we go beyond BRAF in clinics?. J. Cancer Res. Clin. Oncol..

[22] Kainulainen K., Takabe P., Heikkinen S., Aaltonen N., de la Motte C., Rauhala L., Durst F.C., Oikari S., Hukkanen T., Rahunen E. (2022). M1 macrophages induce protumor inflammation in melanoma cells through TNFR-NF-κB signaling. J. Investig. Dermatol..

[23] Bagheri H., Pourhanifeh M.H., Derakhshan M., Mahjoubin-Tehran M., Ghasemi F., Mousavi S., Rafiei R., Abbaszadeh-Goudarzi K., Mirzaei H.R., Mirzaei H. (2020). CXCL-10: A new candidate for melanoma therapy?. Cell. Oncol..

[24] He R., Lu J., Feng J., Lu Z., Shen K., Xu K., Luo H., Yang G., Chi H., Huang S. (2024). Advancing immunotherapy for melanoma: The critical role of single-cell analysis in identifying predictive biomarkers. Front. Immunol..

[25] Reschke R., Deitert B., Enk A.H., Hassel J.C. (2024). The role of tissue-resident memory T cells as mediators for response and toxicity in immunotherapy-treated melanoma—Two sides of the same coin?. Front. Immunol..

[26] Habib S., Osborn G., Willsmore Z., Chew M.W., Jakubow S., Fitzpatrick A., Wu Y., Sinha K., Lloyd-Hughes H., Geh J.L.C. (2024). Tumor-associated macrophages as key contributors and targets in current and future therapies for melanoma. Expert Rev. Clin. Immunol..

[27] Pieniazek M., Matkowski R., Donizy P. (2018). Macrophages in skin melanoma—The key element in melanomagenesis. Oncol. Lett..

[28] Bønnelykke-Behrndtz M.L., Steiniche T., Damsgaard T.E., Georgsen J.B., Danielsen A., Bastholt L., Møller H.J., Nørgaard P.H., Schmidt H. (2015). MelanA-negative spindle-cell associated melanoma, a distinct inflammatory phenotype correlated with dense infiltration of CD163 macrophages and loss of E-cadherin. Melanoma Res..

[29] Nabizadeh J.A., Manthey H.D., Steyn F.J., Chen W., Widiapradja A., Md Akhir F.N., Boyle G.M., Taylor S.M., Woodruff T.M., Rolfe B.E. (2016). The complement C3a receptor contributes to melanoma tumorigenesis by inhibiting neutrophil and CD4+ T cell responses. J. Immunol..

[30] Gerlini G., Susini P., Sestini S., Brandani P., Giannotti V., Borgognoni L. (2024). Langerhans cells in sentinel lymph nodes from melanoma patients. Cancers.

[31] Alvarez-Dominguez C., Calderón-Gonzalez R., Terán-Navarro H., Salcines-Cuevas D., Garcia-Castaño A., Freire J., Gomez-Roman J., Rivera F. (2017). Dendritic cell therapy in melanoma. Ann. Transl. Med..

[32] Wang X., Montoyo-Pujol Y.G., Bermudez S., Corpas G., Martin A., Almazan F., Cabrera T., López-Nevot M.A. (2021). Serum cytokine profiles of melanoma patients and their association with tumor progression and metastasis. J. Oncol..

[33] Chow M.T., Sceneay J., Paget C., Wong C.S., Duret H., Tschopp J., Möller A., Smyth M.J. (2012). NLRP3 suppresses NK cell-mediated responses to carcinogen-induced tumors and metastases. Cancer Res..

[34] Drexler S.K., Bonsignore L., Masin M., Tardivel A., Jackstadt R., Hermeking H., Schneider P., Gross O., Tschopp J., Yazdi A.S. (2012). Tissue-specific opposing functions of the inflammasome adaptor ASC in the regulation of epithelial skin carcinogenesis. Proc. Natl. Acad. Sci. USA.

[35] Theivanthiran B., Haykal T., Cao L., Holtzhausen A., Plebanek M., DeVito N.C., Hanks B.A. (2021). Overcoming immunotherapy resistance by targeting the tumor-intrinsic NLRP3-HSP70 signaling axis. Cancers.

[36] Ahmad I., Muneer K.M., Tamimi I.A., Chang M.E., Ata M.O., Yusuf N. (2013). Thymoquinone suppresses metastasis of melanoma cells by inhibition of NLRP3 inflammasome. Toxicol. Appl. Pharmacol..

[37] Cesati M., Scatozza F., D’Arcangelo D., Antonini-Cappellini G.C., Rossi S., Tabolacci C., Nudo M., Palese E., Lembo L., Di Lella G. (2020). Investigating serum and tissue expression identified a cytokine/chemokine signature as a highly effective melanoma marker. Cancers.

[38] Tengesdal I.W., Menon D.R., Osborne D.G., Neff C.P., Powers N.E., Gamboni F., Mauro A.G., D’Alessandro A., Stefanoni D., Henen M.A. (2021). Targeting tumor-derived NLRP3 reduces melanoma progression by limiting MDSCs expansion. Proc. Natl. Acad. Sci. USA.

[39] Theivanthiran B., Yarla N., Haykal T., Nguyen Y.V., Cao L., Ferreira M., Holtzhausen A., Al-Rohil R., Salama A.K.S., Beasley G.M. (2022). Tumor-intrinsic NLRP3-HSP70-TLR4 axis drives premetastatic niche development and hyperprogression during anti-PD-1 immunotherapy. Sci. Transl. Med..

[40] Okamoto M., Liu W., Luo Y., Tanaka A., Cai X., Norris D.A., Dinarello C.A., Fujita M. (2010). Constitutively active inflammasome in human melanoma cells mediating autoinflammation via caspase-1 processing and secretion of interleukin-1β. J. Biol. Chem..

[41] Manica D., da Silva G.B., Narzetti R.A., Dallagnoll P., da Silva A.P., Marafon F., Cassol J., de Souza Matias L., Zamoner A., de Oliveira Maciel S.F.V. (2024). Curcumin modulates purinergic signaling and inflammatory response in cutaneous metastatic melanoma cells. Purinergic Signal..

[42] da Silva G.B., Manica D., da Silva A.P., Marafon F., Moreno M., Bagatini M.D. (2023). Rosmarinic acid decreases viability, inhibits migration, and modulates expression of apoptosis-related CASP8/CASP3/NLRP3 genes in human metastatic melanoma cells. Chem. Biol. Interact..

[43] Tengesdal I.W., Dinarello A., Powers N.E., Burchill M.A., Joosten L.A.B., Marchetti C., Dinarello C.A. (2021). Tumor NLRP3-derived IL-1β drives the IL-6/STAT3 axis resulting in sustained MDSC-mediated immunosuppression. Front. Immunol..

[44] Wu L.Y., Park S.H., Jakobsson H., Shackleton M., Möller A. (2024). Immune regulation and immune therapy in melanoma: Review with emphasis on CD155 signalling. Cancers.

[45] Huang J., Chan S.C., Ngai C.H., Lok V., Zhang L., Lucero-Prisno D.E., Xu W., Zheng Z.J., Elcarte E., Withers M. (2022). Disease burden, risk factors, and trends of leukemia: A global analysis. Front. Oncol..

[46] Saluja S., Bansal I., Bhardwaj R., Beg M.S., Palanichamy J.K. (2024). Inflammation as a driver of hematological malignancies. Front. Oncol..

[47] Giles F.J., Krawczyk J., O’Dwyer M., Swords R., Freeman C. (2014). The role of inflammation in leukemia. Adv. Exp. Med. Biol..

[48] Bonifant C.L., Tasian S.K. (2020). The future of cellular immunotherapy for childhood leukemia. Curr. Opin. Pediatr..

[49] Inaba H., Pui C.H. (2019). Immunotherapy in pediatric acute lymphoblastic leukemia. Cancer Metastasis Rev..

[50] Jasinski S., De Los Reyes F.A., Yametti G.C., Pierro J., Raetz E., Carroll W.L. (2020). Immunotherapy in pediatric B-cell acute lymphoblastic leukemia: Advances and ongoing challenges. Paediatr. Drugs.

[51] Andina N., Bonadies N., Allam R. (2022). Inflammasome Activation in Myeloid Malignancies-Friend or Foe?. Front. Cell Dev. Biol..

[52] Alves-Hanna F.S., Crespo-Neto J.A., Nogueira G.M., Pereira D.S., Lima A.B., Ribeiro T.L.P., Santos V.G.R., Fonseca J.R.F., Magalhães-Gama F., Sadahiro A. (2023). Insights Regarding the Role of Inflammasomes in Leukemia: What Do We Know?. J. Immunol. Res..

[53] Urwanisch L., Luciano M., Horejs-Hoeck J. (2021). The NLRP3 Inflammasome and Its Role in the Pathogenicity of Leukemia. Int. J. Mol. Sci..

[54] Zhong C., Wang R., Hua M., Zhang C., Han F., Xu M., Yang X., Li G., Hu X., Sun T. (2021). NLRP3 Inflammasome Promotes the Progression of Acute Myeloid Leukemia via IL-1β Pathway. Front. Immunol..

[55] Wang H., Hua M., Wang S., Yu J., Chen C., Zhao X., Zhang C., Zhong C., Wang R., He N. (2017). Genetic Polymorphisms of IL-18 rs1946518 and IL-1β rs16944 Are Associated with Prognosis and Survival of Acute Myeloid Leukemia. Inflamm. Res..

[56] Liu Q., Hua M., Zhang C., Wang R., Liu J., Yang X., Han F., Hou M., Ma D. (2021). NLRP3-Activated Bone Marrow Dendritic Cells Play Antileukemic Roles via IL-1β/Th1/IFN-γ in Acute Myeloid Leukemia. Cancer Lett..

[57] Liu N., Wu Y., Wen X., Li P., Lu F., Shang H. (2021). Chronic Stress Promotes Acute Myeloid Leukemia Progression Through HMGB1/NLRP3/IL-1β Signaling Pathway. J. Mol. Med..

[58] Hamarsheh S., Osswald L., Saller B.S., Unger S., De Feo D., Vinnakota J.M., Konantz M., Uhl F.M., Becker H., Lübbert M. (2020). Oncogenic KrasG12D Causes Myeloproliferation via NLRP3 Inflammasome Activation. Nat. Commun..

[59] Jia Y., Zhang C., Hua M., Wang M., Chen P., Ma D. (2017). Aberrant NLRP3 Inflammasome Associated with Aryl Hydrocarbon Receptor Potentially Contributes to the Imbalance of T-Helper Cells in Patients with Acute Myeloid Leukemia. Oncol. Lett..

[60] Hurtado-Navarro L., Cuenca-Zamora E.J., Zamora L., Bellosillo B., Such E., Soler-Espejo E., Martínez-Banaclocha H., Hernández-Rivas J.M., Marco-Ayala J., Martínez-Alarcón L. (2023). NLRP3 Inflammasome Activation and Symptom Burden in KRAS-Mutated CMML Patients Is Reverted by IL-1 Blocking Therapy. Cell Rep. Med..

[61] Song Z., Wang M., Ge Y., Chen X.P., Xu Z., Sun Y., Xiong X.F. (2021). Tyrosine Phosphatase SHP2 Inhibitors in Tumor-Targeted Therapies. Acta Pharm. Sin. B.

[62] Tomasik J., Basak G.W. (2022). Inflammasomes-New Contributors to Blood Diseases. Int. J. Mol. Sci..

[63] Pourhassan H., Murphy L., Aldoss I. (2024). Glucocorticoid Therapy in Acute Lymphoblastic Leukemia: Navigating Short-Term and Long-Term Effects and Optimal Regimen Selection. Curr. Hematol. Malig. Rep..

[64] Paugh S.W., Bonten E.J., Savic D., Ramsey L.B., Thierfelder W.E., Gurung P., Malireddi R.K., Actis M., Mayasundari A., Min J. (2015). NALP3 Inflammasome Upregulation and CASP1 Cleavage of the Glucocorticoid Receptor Cause Glucocorticoid Resistance in Leukemia Cells. Nat. Genet..

[65] Paugh S.W., Bonten E.J., Evans W.E. (2015). Inflammasome-Mediated Glucocorticoid Resistance: The Receptor Rheostat. Mol. Cell. Oncol..

[66] Hu Z., Sporn M., Letterio J. (2020). Targeting NLRP3 Inflammasome-Induced Therapy Resistance in ALL. Blood.

[67] Singh J., Kumari S., Arora M., Verma D., Palanichamy J.K., Kumar R., Sharma G., Bakhshi S., Pushpam D., Ali M.S. (2021). Prognostic Relevance of Expression of EMP1, CASP1, and NLRP3 Genes in Pediatric B-Lineage Acute Lymphoblastic Leukemia. Front. Oncol..

[68] Alves F.S., Xabregas L.A., Kerr M.W.A., Souza G.L., Pereira D.S., Magalhães-Gama F., Santiago M.R.R., Garcia N.P., Tarragô A.M., Ogusku M.M. (2021). Genetic Polymorphisms of Inflammasome Genes Associated with Pediatric Acute Lymphoblastic Leukemia and Clinical Prognosis in the Brazilian Amazon. Sci. Rep..

[69] Zhang A., Yu J., Yan S., Zhao X., Chen C., Zhou Y., Zhao X., Hua M., Wang R., Zhang C. (2018). The Genetic Polymorphism and Expression Profiles of NLRP3 Inflammasome in Patients with Chronic Myeloid Leukemia. Hum. Immunol..

[70] Adinolfi E., Melchiorri L., Falzoni S., Chiozzi P., Morelli A., Tieghi A., Cuneo A., Castoldi G., Di Virgilio F., Baricordi O.R. (2002). P2X7 Receptor Expression in Evolutive and Indolent Forms of Chronic B Lymphocytic Leukemia. Blood.

[71] Salaro E., Rambaldi A., Falzoni S., Amoroso F.S., Franceschini A., Sarti A.C., Bonora M., Cavazzini F., Rigolin G.M., Ciccone M. (2016). Involvement of the P2X7-NLRP3 Axis in Leukemic Cell Proliferation and Death. Sci. Rep..

[72] Wang X., Lin J., Wang Z., Li Z., Wang M. (2023). Possible Therapeutic Targets for NLRP3 Inflammasome-Induced Breast Cancer. Discov. Oncol..

[73] Sonnessa M., Cioffi A., Brunetti O., Silvestris N., Zito F.A., Saponaro C., Mangia A. (2020). NLRP3 Inflammasome From Bench to Bedside: New Perspectives for Triple Negative Breast Cancer. Front. Oncol..

[74] Faria S.S., Costantini S., de Lima V.C.C., de Andrade V.P., Rialland M., Cedric R., Budillon A., Magalhães K.G. (2021). NLRP3 Inflammasome-Mediated Cytokine Production and Pyroptosis Cell Death in Breast Cancer. J. Biomed. Sci..

[75] Quagliariello V., De Laurentiis M., Cocco S., Rea G., Bonelli A., Caronna A., Lombari M.C., Conforti G., Berretta M., Botti G. (2020). NLRP3 as Putative Marker of Ipilimumab-Induced Cardiotoxicity in the Presence of Hyperglycemia in Estrogen-Responsive and Triple-Negative Breast Cancer Cells. Int. J. Mol. Sci..

[76] Chang C.M., Liang T.R., Lam H.Y.P. (2024). The Use of Schisandrin B to Combat Triple-Negative Breast Cancers by Inhibiting NLRP3-Induced Interleukin-1β Production. Biomolecules.

[77] Holen I., Lefley D.V., Francis S.E., Rennicks S., Bradbury S., Coleman R.E., Ottewell P. (2016). IL-1 Drives Breast Cancer Growth and Bone Metastasis in Vivo. Oncotarget.

[78] Li K., Wei L., Huang Y., Wu Y., Su M., Pang X., Wang N., Ji F., Zhong C., Chen T. (2016). Leptin Promotes Breast Cancer Cell Migration and Invasion via IL-18 Expression and Secretion. Int. J. Oncol..

[79] Zheng Q., Yao D., Cai Y., Zhou T. (2020). NLRP3 Augmented Resistance to Gemcitabine in Triple-Negative Breast Cancer Cells via EMT/IL-1β/Wnt/β-Catenin Signaling Pathway. Biosci. Rep..

[80] Reed J.R., Leon R.P., Hall M.K., Schwertfeger K.L. (2009). Interleukin-1β and Fibroblast Growth Factor Receptor 1 Cooperate to Induce Cyclooxygenase-2 During Early Mammary Tumorigenesis. Breast Cancer Res..

[81] Zhou J., Ottewell P.D. (2024). The Role of IL-1β in Breast Cancer Bone Metastasis. J. Bone Oncol..

[82] Jeon M., Han J., Nam S.J., Lee J.E., Kim S. (2016). Elevated IL-1β Expression Induces Invasiveness of Triple Negative Breast Cancer Cells and Is Suppressed by Zerumbone. Chem. Biol. Interact..

[83] Han J., Bae S.Y., Oh S.J., Lee J., Lee J.H., Lee H.C., Lee S.K., Kil W.H., Kim S.W., Nam S.J. (2014). Zerumbone Suppresses IL-1β-Induced Cell Migration and Invasion by Inhibiting IL-8 and MMP-3 Expression in Human Triple-Negative Breast Cancer Cells. Phytother. Res..

[84] Perez-Yepez E.A., Ayala-Sumuano J.T., Lezama R., Meza I. (2014). A Novel β-Catenin Signaling Pathway Activated by IL-1β Leads to the Onset of Epithelial-Mesenchymal Transition in Breast Cancer Cells. Cancer Lett..

[85] García-Morales L., Mendoza-Rodríguez M.G., Tapia Ramírez J., Meza I. (2023). CBD Inhibits In Vivo Development of Human Breast Cancer Tumors. Int. J. Mol. Sci..

[86] Pham D.V., Raut P.K., Pandit M., Chang J.H., Katila N., Choi D.Y., Jeong J.H., Park P.H. (2020). Globular Adiponectin Inhibits Breast Cancer Cell Growth Through Modulation of Inflammasome Activation: Critical Role of Sestrin2 and AMPK Signaling. Cancers.

[87] Chen Q., Lei J.H., Bao J., Wang H., Hao W., Li L., Peng C., Masuda T., Miao K., Xu J. (2020). BRCA1 Deficiency Impairs Mitophagy and Promotes Inflammasome Activation and Mammary Tumor Metastasis. Adv. Sci..

[88] Zhang L., Li H., Zang Y., Wang F. (2019). NLRP3 Inflammasome Inactivation Driven by miR-223-3p Reduces Tumor Growth and Increases Anticancer Immunity in Breast Cancer. Mol. Med. Rep..

[89] Xia J., Yu X., Tang L., Li G., He T. (2015). P2X7 Receptor Stimulates Breast Cancer Cell Invasion and Migration via the AKT Pathway. Oncol. Rep..

[90] Du Y., Cao Y., Song W., Wang X., Yu Q., Peng X., Zhao R. (2024). Role of the P2X7 Receptor in Breast Cancer Progression. Purinergic Signal..

[91] Yu X., Chen X., Tang X., Cao Y., Tang L., Liu Y. (2020). P2X7 Blockade Inhibits the Growth of Breast Cancer in 4T1 Breast Cancer-Bearing Mice by NLRP3/Caspase 1 Pathway. Arch. Med. Sci..

[92] Thandra K.C., Barsouk A., Saginala K., Aluru J.S., Barsouk A. (2021). Epidemiology of Lung Cancer. Contemp. Oncol..

[93] Volk R.J., Myers R.E., Arenberg D., Caverly T.J., Hoffman R.M., Katki H.A., Mazzone P.J., Moulton B.W., Reuland D.S., Tanner N.T. (2024). The American Cancer Society National Lung Cancer Roundtable Strategic Plan: Current Challenges and Future Directions for Shared Decision Making for Lung Cancer Screening. Cancer.

[94] Liu L., Soler J., Reckamp K.L., Sankar K. (2024). Emerging Targets in Non-Small Cell Lung Cancer. Int. J. Mol. Sci..

[95] Hendriks L.E.L., Remon J., Faivre-Finn C., Garassino M.C., Heymach J.V., Kerr K.M., Tan D.S.W., Veronesi G., Reck M. (2024). Non-Small-Cell Lung Cancer. Nat. Rev. Dis. Primers..

[96] Annareddy S., Ghewade B., Jadhav U., Wagh P., Sarkar S. (2024). Unveiling the Long-Term Lung Consequences of Smoking and Tobacco Consumption: A Narrative Review. Cureus.

[97] Jabeen S., Ahmed N., Rashid F., Lal N., Kong F., Fu Y., Zhang F. (2024). Circular RNAs in Tuberculosis and Lung Cancer. Clin. Chim. Acta.

[98] Ferdosnejad K., Zamani M.S., Soroush E., Fateh A., Siadat S.D., Tarashi S. (2024). Tuberculosis and Lung Cancer: Metabolic Pathways Play a Key Role. Nucleosides Nucleotides Nucleic Acids.

[99] Malhotra J., Malvezzi M., Negri E., La Vecchia C., Boffetta P. (2016). Risk Factors for Lung Cancer Worldwide. Eur. Respir. J..

[100] Xu L., Cao P., Wang J., Zhang P., Hu S., Cheng C., Wang H. (2024). IL-22: A Key Inflammatory Mediator as a Biomarker and Potential Therapeutic Target for Lung Cancer. Heliyon.

[101] Thapa R., Moglad E., Goyal A., Bhat A.A., Almalki W.H., Kazmi I., Alzarea S.I., Ali H., Oliver B.G., MacLoughlin R. (2024). Deciphering NF-kappaB Pathways in Smoking-Related Lung Carcinogenesis. EXCLI J..

[102] Mo R., Zhang J., Chen Y., Ding Y. (2022). Nicotine Promotes Chronic Obstructive Pulmonary Disease via Inducing Pyroptosis Activation in Bronchial Epithelial Cells. Mol. Med. Rep..

[103] Zhang M.Y., Jiang Y.X., Yang Y.C., Liu J.Y., Huo C., Ji X.L., Qu Y.Q. (2021). Cigarette Smoke Extract Induces Pyroptosis in Human Bronchial Epithelial Cells Through the ROS/NLRP3/Caspase-1 Pathway. Life Sci..

[104] Rumora L., Somborac-Bačura A., Hlapčić I., Hulina-Tomašković A., Rajković M.G. (2020). Cigarette Smoke and Extracellular Hsp70 Induce Secretion of ATP and Differential Activation of NLRP3 Inflammasome in Monocytic and Bronchial Epithelial Cells. Cytokine.

[105] Chen Y., Zhang Y., Li N., Jiang Z., Li X. (2023). Role of Mitochondrial Stress and the NLRP3 Inflammasome in Lung Diseases. Inflamm. Res..

[106] Wang G.L., Xu Y.L., Zhao K.M., Sui A.F., Wang L.N., Deng H., Wang G. (2024). Anti-Inflammatory Effects of Tao Hong Si Wu Tang in Mice with Lung Cancer and Chronic Obstructive Pulmonary Disease. World J. Clin. Oncol..

[107] Rao X., Zhou D., Deng H., Chen Y., Wang J., Zhou X., Jie X., Xu Y., Wu Z., Wang G. (2023). Activation of NLRP3 Inflammasome in Lung Epithelial Cells Triggers Radiation-Induced Lung Injury. Respir. Res..

[108] Li H., Zhang J., Yu B., Yang T., Liu B., Li F., Jin X., Li Q. (2024). RSPO3 Regulates the Radioresistance of Non-Small Cell Lung Cancer Cells via NLRP3 Inflammasome-Mediated Pyroptosis. Radiother. Oncol..

[109] Zhang L., Xu C., Chen X., Shi Q., Su W., Zhao H. (2018). SOCS-1 Suppresses Inflammation Through Inhibition of NALP3 Inflammasome Formation in Smoke Inhalation-Induced Acute Lung Injury. Inflammation.

[110] Ye P., Wang X., Ge S., Chen W., Wang W., Han X. (2019). Long-Term Cigarette Smoking Suppresses NLRP3 Inflammasome Activation in Oral Mucosal Epithelium and Attenuates Host Defense Against Candida albicans in a Rat Model. Biomed. Pharmacother..

[111] Wang Y., Kong H., Zeng X., Liu W., Wang Z., Yan X., Wang H., Xie W. (2016). Activation of NLRP3 Inflammasome Enhances the Proliferation and Migration of A549 Lung Cancer Cells. Oncol. Rep..

[112] Dutkowska A., Szmyd B., Kaszkowiak M., Domańska-Senderowska D., Pastuszak-Lewandoska D., Brzeziańska-Lasota E., Kordiak J., Antczak A. (2021). Expression of Inflammatory Interleukins and Selected miRNAs in Non-Small Cell Lung Cancer. Sci. Rep..

[113] Kong H., Wang Y., Zeng X., Wang Z., Wang H., Xie W. (2015). Differential Expression of Inflammasomes in Lung Cancer Cell Lines and Tissues. Tumour Biol..

[114] Huang L., Duan S., Shao H., Zhang A., Chen S., Zhang P., Wang N., Wang W., Wu Y., Wang J. (2019). NLRP3 Deletion Inhibits Inflammation-Driven Mouse Lung Tumorigenesis Induced by Benzo(a)pyrene and Lipopolysaccharide. Respir. Res..

[115] Yano S., Nokihara H., Yamamoto A., Goto H., Ogawa H., Kanematsu T., Miki T., Uehara H., Saijo Y., Nukiwa T. (2003). Multifunctional Interleukin-1β Promotes Metastasis of Human Lung Cancer Cells in SCID Mice via Enhanced Expression of Adhesion-, Invasion- and Angiogenesis-Related Molecules. Cancer Sci..

[116] Li Z., Yang F., Zhu X., Zhou B., Jin K., Dai J., Jiang G. (2024). Effect of NLRP3 Inflammasome on Lung Cancer Immune Microenvironment Activation and Its Mechanism. Altern. Ther. Health Med..

[117] Metwally Y.F., Elsaid A.M., Elsadda R.R., Refaat S., Zahran R.F. (2024). Impact of IL-6 and IL-1β Gene Variants on Non-small-cell Lung Cancer Risk in Egyptian Patients. Biochem. Genet..

[118] Liu W., Xin M., Li Q., Sun L., Han X., Wang J. (2022). IL-17A Promotes the Migration, Invasion and the EMT Process of Lung Cancer Accompanied by NLRP3 Activation. Biomed. Res. Int..

[119] Bodnar-Wachtel M., Huber A.L., Gorry J., Hacot S., Burlet D., Gérossier L., Guey B., Goutagny N., Bartosch B., Ballot E. (2024). NLRP3 and Gasdermin D-Mediated Pyroptosis Contributes to Resistance of Small Cell Lung Cancer to Anticancer Chemotherapy. J. Clin. Investig..

[120] Zhu H., Guan Y., Wang W., Liu X., Wang S., Zheng R., Li Y., Liu L., Huang H. (2024). Reniformin A Suppresses Non-Small Cell Lung Cancer Progression by Inducing TLR4/NLRP3/Caspase-1/GSDMD-Dependent Pyroptosis. Int. Immunopharmacol..

[121] Chen M., Hu C., Yang L., Guo Q., Liang Y., Wang W. (2023). Saikosaponin-D Induces the Pyroptosis of Lung Cancer by Increasing ROS and Activating the NF-κB/NLRP3/Caspase-1/GSDMD Pathway. J. Biochem. Mol. Toxicol..

[122] Zhao X., Chen C., Han W., Liang M., Cheng Y., Chen Y., Pang D., Lei H., Feng X., Cao S. (2024). EEBR Induces Caspase-1-Dependent Pyroptosis Through the NF-κB/NLRP3 Signalling Cascade in Non-Small Cell Lung Cancer. J. Cell. Mol. Med..

[123] Zhao Z., Ma D., Qin Y., Xu Y., Li S., Liu H. (2024). Melatonin Downregulates Angiogenesis and Lymphangiogenesis by Regulating Tumor-Associated Macrophages via NLRP3 Inflammasomes in Lung Adenocarcinoma. Aging.

[124] Morgan E., Arnold M., Gini A., Lorenzoni V., Cabasag C.J., Laversanne M., Vignat J., Ferlay J., Murphy N., Bray F. (2023). Global Burden of Colorectal Cancer in 2020 and 2040: Incidence and Mortality Estimates from GLOBOCAN. Gut.

[125] Brandaleone L., Dal Buono A., Gabbiadini R., Marcozzi G., Polverini D., Carvello M., Spinelli A., Hassan C., Repici A., Bezzio C. (2024). Hereditary Colorectal Cancer Syndromes and Inflammatory Bowel Diseases: Risk Management and Surveillance Strategies. Cancers.

[126] Goudarzi Y., Monirvaghefi K., Aghaei S., Amiri S.S., Rezaei M., Dehghanitafti A., Azarpey A., Azani A., Pakmehr S., Eftekhari H.R. (2024). Effect of Genetic Profiling on Surgical Decisions at Hereditary Colorectal Cancer Syndromes. Heliyon.

[127] Iacucci M., Santacroce G., Majumder S., Morael J., Zammarchi I., Maeda Y., Ryan D., Di Sabatino A., Rescigno M., Aburto M.R. (2024). Opening the Doors of Precision Medicine: Novel Tools to Assess Intestinal Barrier in Inflammatory Bowel Disease and Colitis-Associated Neoplasia. Gut.

[128] Li Y., Li H., Cui M., Zhou Y., Zhang M., Zhang M. (2024). Interaction of Exosomal MicroRNA and Oxidative Stress in the Pathogenesis of Colitis-Associated Cancer. Front. Biosci..

[129] Yu L., Zhang M.M., Hou J.G. (2022). Molecular and Cellular Pathways in Colorectal Cancer: Apoptosis, Autophagy and Inflammation as Key Players. Scand. J. Gastroenterol..

[130] Direito R., Barbalho S.M., Figueira M.E., Minniti G., de Carvalho G.M., de Oliveira Zanuso B., de Oliveira Dos Santos A.R., de Góes Corrêa N., Rodrigues V.D., de Alvares Goulart R. (2023). Medicinal Plants, Phytochemicals and Regulation of the NLRP3 Inflammasome in Inflammatory Bowel Diseases: A Comprehensive Review. Metabolites.

[131] Aggeletopoulou I., Kalafateli M., Tsounis E.P., Triantos C. (2024). Exploring the role of IL-1β in inflammatory bowel disease pathogenesis. Front. Med..

[132] Bank S., Julsgaard M., Abed O.K., Burisch J., Broder Brodersen J., Pedersen N.K., Gouliaev A., Ajan R., Nytoft Rasmussen D., Honore Grauslund C. (2019). Polymorphisms in the NFkB, TNF-Alpha, IL-1Beta, and IL-18 Pathways Are Associated with Response to Anti-TNF Therapy in Danish Patients with Inflammatory Bowel Disease. Aliment. Pharmacol. Ther..

[133] Bauer C., Duewell P., Lehr H.A., Endres S., Schnurr M. (2012). Protective and Aggravating Effects of NLRP3 Inflammasome Activation in IBD Models: Influence of Genetic and Environmental Factors. Dig. Dis..

[134] Bauer C., Duewell P., Mayer C., Lehr H.A., Fitzgerald K.A., Dauer M., Tschopp J., Endres S., Latz E., Schnurr M. (2010). Colitis Induced in Mice with Dextran Sulfate Sodium (DSS) Is Mediated by the NLRP3 Inflammasome. Gut.

[135] Itani S., Watanabe T., Nadatani Y., Sugimura N., Shimada S., Takeda S., Otani K., Hosomi S., Nagami Y., Tanaka F. (2016). NLRP3 Inflammasome Has a Protective Effect Against Oxazolone-Induced Colitis: A Possible Role in Ulcerative Colitis. Sci. Rep..

[136] Guo W., Sun Y., Liu W., Wu X., Guo L., Cai P., Wu X., Wu X., Shen Y., Shu Y. (2014). Small Molecule-Driven Mitophagy-Mediated NLRP3 Inflammasome Inhibition Is Responsible for the Prevention of Colitis-Associated Cancer. Autophagy.

[137] Perera A.P., Kunde D., Eri R. (2017). NLRP3 Inhibitors as Potential Therapeutic Agents for Treatment of Inflammatory Bowel Disease. Curr. Pharm. Des..

[138] Hu D., Li Y., Wang X., Zou H., Li Z., Chen W., Meng Y., Wang Y., Li Q., Liao F. (2024). Palmitoylation of NLRP3 Modulates Inflammasome Activation and Inflammatory Bowel Disease Development. J. Immunol..

[139] Dharmapuri G., Kotha A.K., Kalangi S.K., Reddanna P. (2024). Mangiferin, A Naturally Occurring Glucosylxanthone, Induces Apoptosis in Caco-2 Cells In Vitro and Exerts Protective Effects on Acetic Acid-Induced Ulcerative Colitis in Mice through the Regulation of NLRP3. ACS Pharmacol. Transl. Sci..

[140] Feng S.H., Zhao B., Zhan X., Li R.H., Yang Q., Wang S.M., Li A. (2024). Quercetin-Induced Pyroptosis in Colon Cancer through NEK7-Mediated NLRP3 Inflammasome-GSDMD Signaling Pathway Activation. Am. J. Cancer Res..

[141] Allen I.C., TeKippe E.M., Woodford R.M., Uronis J.M., Holl E.K., Rogers A.B., Herfarth H.H., Jobin C., Ting J.P. (2010). The NLRP3 Inflammasome Functions as a Negative Regulator of Tumorigenesis During Colitis-Associated Cancer. J. Exp. Med..

[142] Chen Y., Ma S., Pi D., Wu Y., Zuo Q., Li C., Ouyang M. (2022). Luteolin Induces Pyroptosis in HT-29 Cells by Activating the Caspase-1/Gasdermin D Signalling Pathway. Front. Pharmacol..

[143] Li X.Y., Yang Y.T., Zhao Y., Kong X.H., Yang G., Hong J., Zhang D., Ma X.P. (2021). Moxibustion Inhibits the Expression of Colonic NLRP3 through miR7/RNF183/NF-κB Signaling Pathway in UC Rats. Evid.-Based Complement. Alternat. Med..

[144] Zhang Z., Li M., Tai Y., Xing Y., Zuo H., Jin X., Ma J. (2024). ZNF70 Regulates IL-1β Secretion of Macrophages to Promote the Proliferation of HCT116 Cells via Activation of NLRP3 Inflammasome and STAT3 Pathway in Colitis-Associated Colorectal Cancer. Cell Signal..

[145] Wang S.L., Zhang M.M., Zhou H., Su G.Q., Ding Y., Xu G.H., Wang X., Li C.F., Huang W.F., Yi L.T. (2023). Inhibition of NLRP3 Attenuates Sodium Dextran Sulfate-Induced Inflammatory Bowel Disease through Gut Microbiota Regulation. Biomed. J..

[146] Gao Q., Tian W., Yang H., Hu H., Zheng J., Yao X., Hu B., Liu H. (2024). Shen-Ling-Bai-Zhu-San Alleviates the Imbalance of Intestinal Homeostasis in Dextran Sodium Sulfate-Induced Colitis Mice by Regulating Gut Microbiota and Inhibiting the NLRP3 Inflammasome Activation. J. Ethnopharmacol..

[147] Zhen Y., Zhang H. (2019). NLRP3 Inflammasome and Inflammatory Bowel Disease. Front. Immunol..

[148] Wang P., Gu Y., Yang J., Qiu J., Xu Y., Xu Z., Gao J., Wan C. (2022). The Prognostic Value of NLRP1/NLRP3 and Its Relationship with Immune Infiltration in Human Gastric Cancer. Aging.

[149] Zhang K., Lin G., Nie Z., Jin S., Bing X., Li Z., Li M. (2024). TRIM38 Suppresses Migration, Invasion, Metastasis, and Proliferation in Non-Small Cell Lung Cancer (NSCLC) via Regulating the AMPK/NF-κB/NLRP3 Pathway. Mol. Cell. Biochem..

[150] Zhang L., Wang Y., Liu X., Zhang Y. (2022). NLRP3 Inflammasome Activation in MΦs-CRC Crosstalk Promotes Colorectal Cancer Metastasis. Ann. Clin. Lab. Sci..

[151] Deng Q., Geng Y., Zhao L., Li R., Zhang Z., Li K., Liang R., Shao X., Huang M., Zuo D. (2019). NLRP3 Inflammasomes in Macrophages Drive Colorectal Cancer Metastasis to the Liver. Cancer Lett..

[152] Inoue M. (2024). Epidemiology of Gastric Cancer—Changing Trends and Global Disparities. Cancers.

[153] Lin Y., Liu K., Lu F., Zhai C., Cheng F. (2024). Programmed Cell Death in *Helicobacter pylori* Infection and Related Gastric Cancer. Front. Cell. Infect. Microbiol..

[154] Shah D., Bentrem D. (2024). Environmental and Genetic Risk Factors for Gastric Cancer. Cancer Treat. Res..

[155] Shen G., Wang Q., Li Z., Xie J., Han X., Wei Z., Zhang P., Zhao S., Wang X., Huang X. (2024). Bridging Chronic Inflammation and Digestive Cancer: The Critical Role of Innate Lymphoid Cells in Tumor Microenvironments. Int. J. Biol. Sci..

[156] Yuan X.Y., Zhang Y., Zhao X., Chen A., Liu P. (2023). IL-1β, an Important Cytokine Affecting Helicobacter pylori-Mediated Gastric Carcinogenesis. Microb. Pathog..

[157] Hong J.B., Zuo W., Wang A.J., Lu N.H. (2016). *Helicobacter pylori* Infection Synergistic with IL-1β Gene Polymorphisms Potentially Contributes to the Carcinogenesis of Gastric Cancer. Int. J. Med. Sci..

[158] Sun C.C., Li L., Tao H.Q., Jiang Z.C., Wang L., Wang H.J. (2022). The Role of NLRP3 Inflammasome in Digestive System Malignancy. Front. Cell Dev. Biol..

[159] Li L., Liao A. (2023). Application of Pyroptosis Score in the Treatment and Prognosis Evaluation of Gastric Cancer. Zhong Nan Da Xue Xue Bao Yi Xue Ban.

[160] Kumar S., Dhiman M. (2024). *Helicobacter pylori* Secretary Proteins-Induced Oxidative Stress and Its Role in NLRP3 Inflammasome Activation. Cell. Immunol..

[161] Zhang X., Li C., Chen D., He X., Zhao Y., Bao L., Wang Q., Zhou J., Xie Y. (2022). *H. pylori* CagA Activates the NLRP3 Inflammasome to Promote Gastric Cancer Cell Migration and Invasion. Inflamm. Res..

[162] Pachathundikandi S.K., Blaser N., Bruns H., Backert S. (2020). *Helicobacter pylori* Avoids the Critical Activation of NLRP3 Inflammasome-Mediated Production of Oncogenic Mature IL-1β in Human Immune Cells. Cancers.

[163] Jang A.R., Kang M.J., Shin J.I., Kwon S.W., Park J.Y., Ahn J.H., Lee T.S., Kim D.Y., Choi B.G., Seo M.W. (2020). Unveiling the Crucial Role of Type IV Secretion System and Motility of *Helicobacter pylori* in IL-1β Production via NLRP3 Inflammasome Activation in Neutrophils. Front. Immunol..

[164] Banerjee S., Bond J.S. (2008). Prointerleukin-18 is Activated by Meprin Beta in Vitro and in Vivo in Intestinal Inflammation. J. Biol. Chem..

[165] Chen D., Wu L., Liu X., Wang Q., Gui S., Bao L., Wang Z., He X., Zhao Y., Zhou J. (2024). *Helicobacter pylori* CagA-Mediated Mitophagy to Attenuate the NLRP3 Inflammasome Activation and Enhance the Survival of Infected Cells. Sci. Rep..

[166] Qi X., Liu J., Wang L., Gu P., Song S., Shu P. (2024). Kaempferol-Induced Mitochondrial Damage Promotes NF-κB-NLRP3-Caspase-1 Signaling Axis-Mediated Pyroptosis in Gastric Cancer Cells. Heliyon.

[167] Liu J., Qi X., Gu P., Wang L., Song S., Shu P. (2024). Baicalin Induces Gastric Cancer Cell Pyroptosis through the NF-κB-NLRP3 Signaling Axis. J. Cancer.

[168] Che Y., Xu W., Ding C., He T., Xu X., Shuai Y., Huang H., Wu J., Wang Y., Wang C. (2023). Bile Acids Target Mitofusin 2 to Differentially Regulate Innate Immunity in Physiological Versus Cholestatic Conditions. Cell Rep..

[169] Zhao C., Mu M., Li X., Dong Z., Wang J., Yao C., Zheng J., Sun X., Yu J. (2024). USP50 Regulates NLRP3 Inflammasome Activation in Duodenogastric Reflux-Induced Gastric Tumorigenesis. Front. Immunol..

[170] Xu P.P., Wu J., Zhang J., Yu T.Y., Wang Y.B. (2024). Paclitaxel May Inhibit Migration and Invasion of Gastric Cancer Cells via Nod-Like Receptor Family Pyrin Domain-Containing 3/Caspase-1/Gasdermin E Mediated Pyroptosis Pathway. Chem. Biol. Drug Des..

[171] Ren N., Jiang T., Wang C., Xie S., Xing Y., Piao D., Zhang T., Zhu Y. (2020). LncRNA ADAMTS9-AS2 Inhibits Gastric Cancer (GC) Development and Sensitizes Chemoresistant GC Cells to Cisplatin by Regulating miR-223-3p/NLRP3 Axis. Aging.

[172] Schulz C., Koch N., Schütte K., Pieper D.H., Malfertheiner P. (2015). *H. pylori* and Its Modulation of Gastrointestinal Microbiota. J. Dig. Dis..

[173] Andersson A.F., Lindberg M., Jakobsson H., Bäckhed F., Nyrén P., Engstrand L. (2008). Comparative Analysis of Human Gut Microbiota by Barcoded Pyrosequencing. PLoS ONE.

[174] Ruff S.M., Pawlik T.M. (2024). Molecular Classification and Pathogenesis of Pancreatic Adenocarcinoma and Targeted Therapies: A Review. Front. Biosci..

[175] Döppler H.R., Storz P. (2024). Macrophage-Induced Reactive Oxygen Species in the Initiation of Pancreatic Cancer: A Mini-Review. Front. Immunol..

[176] Feng W., Niu N., Lu P., Chen Z., Rao H., Zhang W., Ma C., Liu C., Xu Y., Gao W.Q. (2024). Multilevel Regulation of NF-κB Signaling by NSD2 Suppresses Kras-Driven Pancreatic Tumorigenesis. Adv. Sci..

[177] Fraile-Martinez O., García-Montero C., Pekarek L., Saz J.V., Álvarez-Mon M.Á., Barrena-Blázquez S., García-Honduvilla N., Buján J., Asúnsolo Á., Coca S. (2024). Decreased Survival in Patients with Pancreatic Cancer May Be Associated with an Increase in Histopathological Expression of Inflammasome Marker NLRP3. Histol. Histopathol..

[178] Xu B., Bai B., Sha S., Yu P., An Y., Wang S., Kong X., Liu C., Wei N., Feng Q. (2014). Interleukin-1β Induces Autophagy by Affecting Calcium Homeostasis and Trypsinogen Activation in Pancreatic Acinar Cells. Int. J. Clin. Exp. Pathol..

[179] Caronni N., La Terza F., Vittoria F.M., Barbiera G., Mezzanzanica L., Cuzzola V., Barresi S., Pellegatta M., Canevazzi P., Dunsmore G. (2023). IL-1β+ Macrophages Fuel Pathogenic Inflammation in Pancreatic Cancer. Nature.

[180] Chen Q., Wang J., Zhang Q., Zhang J., Lou Y., Yang J., Chen Y., Wei T., Zhang J., Fu Q. (2019). Tumour Cell-Derived Debris and IgG Synergistically Promote Metastasis of Pancreatic Cancer by Inducing Inflammation via Tumour-Associated Macrophages. Br. J. Cancer.

[181] Wu X., Yang Z., Wang H., Zhao Y., Gao X., Zang B. (2021). High-Mobility Group Box Protein-1 Induces Acute Pancreatitis through Activation of Neutrophil Extracellular Trap and Subsequent Production of IL-1β. Life Sci..

[182] Miskiewicz A., Szparecki G., Durlik M., Rydzewska G., Ziobrowski I., Górska R. (2015). The Q705K and F359L Single-Nucleotide Polymorphisms of NOD-Like Receptor Signaling Pathway: Association with Chronic Pancreatitis, Pancreatic Cancer, and Periodontitis. Arch. Immunol. Ther. Exp..

[183] Boone B.A., Murthy P., Miller-Ocuin J.L., Liang X., Russell K.L., Loughran P., Gawaz M., Lotze M.T., Zeh H.J., Vogel S. (2019). The Platelet NLRP3 Inflammasome is Upregulated in a Murine Model of Pancreatic Cancer and Promotes Platelet Aggregation and Tumor Growth. Ann. Hematol..

[184] Liu H., Xu Y., Liang K., Liu R. (2020). Immune Cells Combined with NLRP3 Inflammasome Inhibitor Exert Better Antitumor Effect on Pancreatic Ductal Adenocarcinoma. Front. Oncol..

[185] Kong X., Lu A.L., Yao X.M., Hua Q., Li X.Y., Qin L., Zhang H.M., Meng G.X., Su Q. (2017). Activation of NLRP3 Inflammasome by Advanced Glycation End Products Promotes Pancreatic Islet Damage. Oxidative Med. Cell. Longev..

[186] Ren Z., Li H., Zhang M., Zhao Y., Fang X., Li X., Chen W., Zhang H., Wang Y., Pan L.L. (2018). A Novel Derivative of the Natural Product Danshensu Suppresses Inflammatory Responses to Alleviate Caerulein-Induced Acute Pancreatitis. Front. Immunol..

[187] Sheng L.P., Han C.Q., Ling X., Guo X.W., Lin R., Ding Z. (2023). Proanthocyanidins Suppress NLRP3 Inflammasome and M1 Macrophage Polarization to Alleviate Severe Acute Pancreatitis in Mice. J. Biochem. Mol. Toxicol..

[188] Xu C., Pascual-Sabater S., Fillat C., Goel A. (2024). The LAMB3-EGFR Signaling Pathway Mediates Synergistic Anti-Cancer Effects of Berberine and Emodin in Pancreatic Cancer. Biochem. Pharmacol..

[189] Ali B.M., Al-Mokaddem A.K., Selim H.M.R.M., Alherz F.A., Saleh A., Hamdan A.M.E., Ousman M.S., El-Emam S.Z. (2024). Pinocembrin’s Protective Effect Against Acute Pancreatitis in a Rat Model: The Correlation Between TLR4/NF-κB/NLRP3 and miR-34a-5p/SIRT1/Nrf2/HO-1 Pathways. Biomed. Pharmacother..

[190] Fawzy H.A., Mohammed A.A., Fawzy H.M., Fikry E.M. (2022). Reorienting of Pramipexole as a Promising Therapy for Acute Pancreatitis in a Rat Model by Suppressing TLR4\NF-κB p65\NLRP3 Inflammasome Signaling. Can. J. Physiol. Pharmacol..

[191] He R., Ye Y., Zhu Q., Xie C. (2024). Systemic Immune-Inflammation Index is Associated with High Risk for Prostate Cancer Among the U.S. Elderly: Evidence from NHANES 2001-2010. Front. Oncol..

[192] Li W., Wang J. (2024). The Current State of Inflammation-Related Research in Prostate Cancer: A Bibliometric Analysis and Systematic Review. Front. Oncol..

[193] Ponomareva L., Liu H., Duan X., Dickerson E., Shen H., Panchanathan R., Choubey D. (2013). AIM2, an IFN-Inducible Cytosolic DNA Sensor, in the Development of Benign Prostate Hyperplasia and Prostate Cancer. Mol. Cancer Res..

[194] Panchanathan R., Liu H., Choubey D. (2016). Hypoxia Primes Human Normal Prostate Epithelial Cells and Cancer Cell Lines for the NLRP3 and AIM2 Inflammasome Activation. Oncotarget.

[195] Xu Z., Wang H., Qin Z., Zhao F., Zhou L., Xu L., Jia R. (2021). NLRP3 Inflammasome Promoted the Malignant Progression of Prostate Cancer via the Activation of Caspase-1. Cell Death Discov..

[196] Zhu M., Liu D., Liu G., Zhang M., Pan F. (2023). Caspase-Linked Programmed Cell Death in Prostate Cancer: From Apoptosis, Necroptosis, and Pyroptosis to PANoptosis. Biomolecules.

[197] Karan D., Tawfik O., Dubey S. (2017). Expression Analysis of Inflammasome Sensors and Implication of NLRP12 Inflammasome in Prostate Cancer. Sci. Rep..

[198] Zeng Y., Li M.X., Wu S.Q., Xu C. (2024). Carvedilol Induces Pyroptosis Through NLRP3-Caspase1-ASC Inflammasome by Nuclear Migration of NF-κB in Prostate Cancer Models. Mol. Biol. Rep..

[199] Gharieb K., Doumandji N., Bensalem W., Bellon R.P., Inoubli L., Siddeek B., Traverse-Glehen A., Decaussin-Petrucci M., Trabucchi M., Benahmed M. (2024). Combined developmental exposure to estrogenic endocrine disruptor and nutritional imbalance induces long term adult prostate inflammation through inflammasome activation. Toxicol. Lett..

[200] Miyauchi T., Narita S., Saiki Y., Kudo-Asabe Y., Horii A., Fukushige S., Habuchi T., Nanjo H., Goto A. (2024). Association Between NLRP3 Inflammasome and Tumor-Node-Metastasis Staging in Prostate Cancer: Immunohistochemical Studies of Prostate Needle Biopsy and Radical Prostatectomy Specimens. Tohoku J. Exp. Med..

[201] Chaudagar K., Rameshbabu S., Mei S., Hirz T., Hu Y.M., Argulian A., Labadie B., Desai K., Grimaldo S., Kahramangil D. (2023). Androgen Blockade Primes NLRP3 in Macrophages to Induce Tumor Phagocytosis. bioRxiv.

[202] Zhao A.N., Yang Z., Wang D.D., Shi B., Zhang H., Bai Y., Yan B.W., Zhang Y., Wen J.K., Wang X.L. (2022). Disturbing NLRP3 Acetylation and Inflammasome Assembly Inhibits Androgen Receptor-Promoted Inflammatory Responses and Prostate Cancer Progression. FASEB J..

[203] Liu Z., Zhu X., Xu C., Min F., Yu G., Chen C. (2023). Ulinastatin Ameliorates the Malignant Progression of Prostate Cancer Cells by Blocking the RhoA/ROCK/NLRP3 Pathway. Drug Dev. Res..

[204] Alsaadi M., Tezcan G., Garanina E.E., Hamza S., McIntyre A., Rizvanov A.A., Khaiboullina S.F. (2021). Doxycycline Attenuates Cancer Cell Growth by Suppressing NLRP3-Mediated Inflammation. Pharmaceuticals.

[205] Mezzasoma L., Talesa V.N., Costanzi E., Bellezza I. (2021). Natriuretic Peptides Regulate Prostate Cells Inflammatory Behavior: Potential Novel Anticancer Agents for Prostate Cancer. Biomolecules.

[206] Giannella L., Ciavattini A. (2023). Screening and Early Diagnosis in Gynecological Cancers. Cancers.

[207] Rathod S., Shanoo A., Acharya N. (2024). Endometriosis: A Comprehensive Exploration of Inflammatory Mechanisms and Fertility Implications. Cureus.

[208] Reis J.L., Rosa N.N., Martins C., Ângelo-Dias M., Borrego L.M., Lima J. (2024). The Role of NK and T Cells in Endometriosis. Int. J. Mol. Sci..

[209] Zhou F., Zhao F., Huang Q., Lin X., Zhang S., Dai Y. (2022). NLRP3 Activated Macrophages Promote Endometrial Stromal Cells Migration in Endometriosis. J. Reprod. Immunol..

[210] Guo X., Xu X., Li T., Yu Q., Wang J., Chen Y., Ding S., Zhu L., Zou G., Zhang X. (2021). NLRP3 Inflammasome Activation of Mast Cells by Estrogen via the Nuclear-Initiated Signaling Pathway Contributes to the Development of Endometriosis. Front. Immunol..

[211] Zhang M., Shi Z., Peng X., Cai D., Peng R., Lin Y., Dai L., Li J., Chen Y., Xiao J. (2023). NLRP3 Inflammasome-Mediated Pyroptosis Induces Notch Signal Activation in Endometriosis Angiogenesis. Mol. Cell. Endocrinol..

[212] Li L., Ye K., Wang D. (2023). Upregulation of HTRA1 Mediated by the lncRNA NEAT1/miR-141-3p Axis Contributes to Endometriosis Development through Activating NLRP3 Inflammasome-Mediated Pyroptotic Cell Death and Cellular Inflammation. In Vitr. Cell. Dev. Biol. Anim..

[213] Hang Y., Tan L., Chen Q., Liu Q., Jin Y. (2021). E3 Ubiquitin Ligase TRIM24 Deficiency Promotes NLRP3/Caspase-1/IL-1β-Mediated Pyroptosis in Endometriosis. Cell Biol. Int..

[214] Fonseca B.M., Pinto B., Costa L., Felgueira E., Rebelo I. (2023). Increased Expression of NLRP3 Inflammasome Components in Granulosa Cells and Follicular Fluid Interleukin(IL)-1β and IL-18 Levels in Fresh IVF/ICSI Cycles in Women with Endometriosis. J. Assist. Reprod. Genet..

[215] Huang Y., Li R., Hu R., Yao J., Yang Y. (2022). PEG2-Induced Pyroptosis Regulates the Expression of HMGB1 and Promotes hEM15A Migration in Endometriosis. Int. J. Mol. Sci..

[216] Arangia A., Marino Y., Fusco R., Siracusa R., Cordaro M., D’Amico R., Macrì F., Raffone E., Impellizzeri D., Cuzzocrea S. (2023). Fisetin, a Natural Polyphenol, Ameliorates Endometriosis Modulating Mast Cells Derived NLRP-3 Inflammasome Pathway and Oxidative Stress. Int. J. Mol. Sci..

[217] Di Nicuolo F., Castellani R., De Cicco Nardone A., Barbaro G., Paciullo C., Pontecorvi A., Scambia G., Di Simone N. (2021). Alpha-Lipoic Acid Plays a Role in Endometriosis: New Evidence on Inflammasome-Mediated Interleukin Production, Cellular Adhesion, and Invasion. Molecules.

[218] Webb P.M., Jordan S.J. (2024). Global Epidemiology of Epithelial Ovarian Cancer. Nat. Rev. Clin. Oncol..

[219] Liu C., Huang X., Su H. (2022). The Role of the Inflammasome and Its Related Pathways in Ovarian Cancer. Clin. Transl. Oncol..

[220] Chang C.M., Wang M.L., Lu K.H., Yang Y.P., Juang C.M., Wang P.H., Hsu R.J., Yu M.H., Chang C.C. (2017). Integrating the Dysregulated Inflammasome-Based Molecular Functionome in the Malignant Transformation of Endometriosis-Associated Ovarian Carcinoma. Oncotarget.

[221] Luborsky J., Barua A., Edassery S., Bahr J.M., Edassery S.L. (2020). Inflammasome Expression is Higher in Ovarian Tumors Than in Normal Ovary. PLoS ONE.

[222] Solini A., Cobuccio L., Rossi C., Parolini F., Biancalana E., Cosio S., Chiarugi M., Gadducci A. (2022). Molecular Characterization of Peritoneal Involvement in Primary Colon and Ovary Neoplasm: The Possible Clinical Meaning of the P2X7 Receptor-Inflammasome Complex. Eur. Surg. Res..

[223] Ding Y., Yan Y., Dong Y., Xu J., Su W., Shi W., Zou Q., Yang X. (2022). NLRP3 Promotes Immune Escape by Regulating Immune Checkpoints: A Pan-Cancer Analysis. Int. Immunopharmacol..

[224] Murakami M., Osuka S., Muraoka A., Hayashi S., Bayasula, Kasahara Y., Sonehara R., Hariyama Y., Shinjo K., Tanaka H. (2022). Effectiveness of NLRP3 Inhibitor as a Non-Hormonal Treatment for Ovarian Endometriosis. Reprod. Biol. Endocrinol..

[225] Wang X., Yin Y., Qian W., Peng C., Shen S., Wang T., Zhao S. (2022). Citric Acid of Ovarian Cancer Metabolite Induces Pyroptosis via the Caspase-4/TXNIP-NLRP3-GSDMD Pathway in Ovarian Cancer. FASEB J..

[226] Li W., Zhao X., Zhang R., Xie J., Zhang G. (2023). Silencing of NLRP3 Sensitizes Chemoresistant Ovarian Cancer Cells to Cisplatin. Mediators Inflamm..

[227] Wu J., Wu Y., Zhao T., Wang X., Guo Q., Wang S., Chen S., Ju X., Li J., Wu X. (2024). Targeting RAC1 Reactivates Pyroptosis to Reverse Paclitaxel Resistance in Ovarian Cancer by Suppressing P21-Activated Kinase 4. MedComm.

[228] Pontillo A., Bricher P., Leal V.N., Lima S., Souza P.R., Crovella S. (2016). Role of Inflammasome Genetics in Susceptibility to HPV Infection and Cervical Cancer Development. J. Med. Virol..

[229] Abdul-Sater A.A., Koo E., Häcker G., Ojcius D.M. (2009). Inflammasome-Dependent Caspase-1 Activation in Cervical Epithelial Cells Stimulates Growth of the Intracellular Pathogen *Chlamydia trachomatis*. J. Biol. Chem..

[230] Lu Q., Lao X., Gan J., Du P., Zhou Y., Nong W., Yang Z. (2023). Impact of NLRP3 Gene Polymorphisms (rs10754558 and rs10733113) on HPV Infection and Cervical Cancer in Southern Chinese Population. Infect. Agent. Cancer.

[231] He A., Shao J., Zhang Y., Lu H., Wu Z., Xu Y. (2017). CD200Fc Reduces LPS-Induced IL-1β Activation in Human Cervical Cancer Cells by Modulating TLR4-NF-κB and NLRP3 Inflammasome Pathway. Oncotarget.

[232] Yu S., Zhao N., He M., Zhang K., Bi X. (2020). MiRNA-214 Promotes the Pyroptosis and Inhibits the Proliferation of Cervical Cancer Cells via Regulating the Expression of NLRP3. Cell. Mol. Biol..

[233] You L., Cui H., Zhao F., Sun H., Zhong H., Zhou G., Chen X. (2021). Inhibition of HMGB1/RAGE Axis Suppressed the Lipopolysaccharide (LPS)-Induced Vicious Transformation of Cervical Epithelial Cells. Bioengineered.

[234] Wu H., Liu J., Zhang Y., Li Q., Wang Q., Gu Z. (2021). miR-22 Suppresses Cell Viability and EMT of Ovarian Cancer Cells via NLRP3 and Inhibits PI3K/AKT Signaling Pathway. Clin. Transl. Oncol..

[235] Fernandes F.P., Cambui R.A.G., Soares J.L.D.S., Reis E.C.D., Leal V.N.C., Pontillo A. (2023). Cervical Carcinoma Induces NLRP3 Inflammasome Activation and IL-1β Release in Human Peripheral Blood Monocytes Affecting Patients’ Overall Survival. Clin. Transl. Oncol..

[236] Ji W., Jin Y., Jiang W. (2024). Foxm1-Mediated Transcriptional Inactivation of NLRP3 Inflammasome Promotes Immunosuppression in Cervical Cancer. Crit. Rev. Eukaryot. Gene Expr..

[237] Barsouk A., Aluru J.S., Rawla P., Saginala K., Barsouk A. (2023). Epidemiology, Risk Factors, and Prevention of Head and Neck Squamous Cell Carcinoma. Med. Sci..

[238] Li K., Zeng X., Liu P., Zeng X., Lv J., Qiu S., Zhang P. (2023). The Role of Inflammation-Associated Factors in Head and Neck Squamous Cell Carcinoma. J. Inflamm. Res..

[239] Bae J.Y., Lee S.W., Shin Y.H., Lee J.H., Jahng J.W., Park K. (2017). P2X7 Receptor and NLRP3 Inflammasome Activation in Head and Neck Cancer. Oncotarget.

[240] Huang C.F., Chen L., Li Y.C., Wu L., Yu G.T., Zhang W.F., Sun Z.J. (2017). NLRP3 Inflammasome Activation Promotes Inflammation-Induced Carcinogenesis in Head and Neck Squamous Cell Carcinoma. J. Exp. Clin. Cancer Res..

[241] Chen L., Huang C.F., Li Y.C., Deng W.W., Mao L., Wu L., Zhang W.F., Zhang L., Sun Z.J. (2018). Blockage of the NLRP3 Inflammasome by MCC950 Improves Anti-Tumor Immune Responses in Head and Neck Squamous Cell Carcinoma. Cell. Mol. Life Sci..

[242] Chen L., Wan S.C., Mao L., Huang C.F., Bu L.L., Sun Z.J. (2023). NLRP3 in Tumor-Associated Macrophages Predicts a Poor Prognosis and Promotes Tumor Growth in Head and Neck Squamous Cell Carcinoma. Cancer Immunol. Immunother..

[243] Jain T., Chandra A., Mishra S.P., Khairnar M., Rajoria S., Maheswari R., Keerthika R., Tiwari S., Agrawal R. (2024). Unravelling the Significance of NLRP3 and IL-1β in Oral Squamous Cell Carcinoma and Potentially Malignant Oral Disorders: A Diagnostic and Prognostic Exploration. Head Neck Pathol..

[244] Yu S., Yin J.J., Miao J.X., Li S.G., Huang C.Z., Huang N., Fan T.L., Li X.N., Wang Y.H., Han S.N. (2020). Activation of NLRP3 Inflammasome Promotes the Proliferation and Migration of Esophageal Squamous Cell Carcinoma. Oncol. Rep..

[245] Feng X., Luo Q., Zhang H., Wang H., Chen W., Meng G., Chen F. (2017). The Role of NLRP3 Inflammasome in 5-Fluorouracil Resistance of Oral Squamous Cell Carcinoma. J. Exp. Clin. Cancer Res..

[246] Yang M., Luo Q., Chen X., Chen F. (2021). Bitter Melon Derived Extracellular Vesicles Enhance the Therapeutic Effects and Reduce the Drug Resistance of 5-Fluorouracil on Oral Squamous Cell Carcinoma. J. Nanobiotechnol..

[247] Chen X., Luo Q., Ding J., Yang M., Zhang R., Chen F. (2020). Zymosan Promotes Proliferation, *Candida albicans* Adhesion and IL-1β Production of Oral Squamous Cell Carcinoma In Vitro. Infect. Agent. Cancer.

[248] Zhang M.J., Gao W., Liu S., Siu S.P., Yin M., Ng J.C., Chow V.L., Chan J.Y., Wong T.S. (2020). CD38 Triggers Inflammasome-Mediated Pyroptotic Cell Death in Head and Neck Squamous Cell Carcinoma. Am. J. Cancer Res..

[249] Xiao L., Li X., Cao P., Fei W., Zhou H., Tang N., Liu Y. (2022). Interleukin-6 Mediated Inflammasome Activation Promotes Oral Squamous Cell Carcinoma Progression via JAK2/STAT3/Sox4/NLRP3 Signaling Pathway. J. Exp. Clin. Cancer Res..

[250] Casili G., Scuderi S.A., Lanza M., Filippone A., Mannino D., Giuffrida R., Colarossi C., Mare M., Capra A.P., De Gaetano F. (2023). Therapeutic Potential of BAY-117082, a Selective NLRP3 Inflammasome Inhibitor, on Metastatic Evolution in Human Oral Squamous Cell Carcinoma (OSCC). Cancers.

[251] Wang L., Wang C., Tao Z., Zhu W., Su Y., Choi W.S. (2023). Tumor-Associated Macrophages Facilitate Oral Squamous Cell Carcinomas Migration and Invasion by MIF/NLRP3/IL-1β Circuit: A Crosstalk Interrupted by Melatonin. Biochim. Biophys. Acta Mol. Basis Dis..

[252] Mishra S.R., Behera B.P., Singh V.K., Mahapatra K.K., Mundkinajeddu D., Bhat D., Minz A.M., Sethi G., Efferth T., Das S. (2024). Anticancer Activity of *Bacopa monnieri* Through Apoptosis Induction and Mitophagy-Dependent NLRP3 Inflammasome Inhibition in Oral Squamous Cell Carcinoma. Phytomedicine.

[253] Yang H.L., Chang C.W., Vadivalagan C., Pandey S., Chen S.J., Lee C.C., Hseu J.H., Hseu Y.C. (2024). Coenzyme Q0 Inhibited the NLRP3 Inflammasome, Metastasis/EMT, and Warburg Effect by Suppressing Hypoxia-Induced HIF-1α Expression in HNSCC Cells. Int. J. Biol. Sci..

